# Ultra‐Stable Aqueous Zinc Anodes: Enabling High‐Performance Zinc‐Ion Batteries via a ZnSiF_6_‐Derived Protective Interphase

**DOI:** 10.1002/advs.202407201

**Published:** 2024-10-07

**Authors:** Yongfeng Huang, Rongsheng Guo, Zejian Li, Jiajia Zhang, Wenbao Liu, Feiyu Kang

**Affiliations:** ^1^ Institute of Materials Research Tsinghua Shenzhen International Graduate School Tsinghua University Shenzhen 518055 China; ^2^ School of Materials Science and Engineering Tsinghua University Beijing 100084 China; ^3^ School of Environmental and Materials Engineering Yantai University Yantai 264005 China

**Keywords:** aqueous zinc‐ion batteries, solid‐electrolyte interphase, zinc anodes, ZnSiF_6_ electrolyte additive

## Abstract

Zinc‐ion batteries (ZIBs) hold immense promise as next‐generation energy storage solutions, however, the practical application of zinc anodes is hindered by dendrite formation and parasitic side reactions. Engineering a stable solid‐ eletrolyte interphase (SEI) is crucial for addressing these issues. This study proposes a novel strategy to enhance Zn anode performance by incorporating a ZnSiF_6_ additive into a standard ZnSO_4_ (ZSO) electrolyte. The ZnSiF_6_ additive facilitates the formation of a stable, fluorine‐rich SEI on the Zn anode surface. Characterization reveals a hierarchical SEI structure, primarily composed of porous alkali zinc sulfate (ZHS) with embedded ZnF_2_. This unique architecture promotes rapid zinc ion desolvation and efficient transport, enhances corrosion resistance, and mitigates hydrogen evolution. Consequently, ZnSiF_6_‐modified cells exhibit exceptional cycling stability, exceeding 3000 hours at 0.5 mA cm^−2^ and 560 hours at 10 mA cm^−2^, significantly outperforming ZSO‐based cells. The modified cells also achieve high areal capacities (10 mAh cm^−2^), indicating superior zinc utilization. This work provides key insights for designing stable electrode/electrolyte interfaces, contributing to the development of high‐performance aqueous ZIBs.

## Graphical abstract

A ZnSiF_6_ additive in ZnSO_4_ (ZSO) electrolyte forms a fluorine‐rich, hierarchical solid‐electrolyte interphase (SEI) on Zn anodes, enhancing Zn‐ion battery (ZIB) performance. The SEI promotes uniform Zn deposition and suppresses side reactions, boosting cycling stability. Characterization reveals a porous ZHS framework with embedded ZnF_2_ within the SEI, contributing to high‐performance aqueous ZIBs.

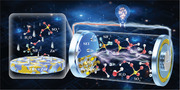

## 1 Introduction

Zinc‐ion batteries (ZIBs) have garnered considerable attention as a promising energy storage technology due to their cost‐effectiveness, environmental benignity, high specific capacity (820 mAh g^−1^ and 5855 mAh cm^−3^), and inherently safe operational characteristics.^[^ ^1^ ^]^ However, the practical implementation of ZIBs is challenged by inherent limitations associated with the zinc anode. These include dendrite growth, detrimental side reactions (e.g., hydrogen evolution reaction), and surface passivation, which ultimately lead to reduced cycling stability and diminished performance.^[^ ^2^ ^]^ Recent research efforts have focused on overcoming these limitations by developing stable solid‐electrolyte interphases (SEIs) on the surface of the Zn anode. The SEI, acting as a critical passivation layer, effectively prevents direct contact between the zinc metal and the electrolyte. This protective barrier plays a crucial role in inhibiting corrosion, suppressing dendrite formation, and mitigating undesirable side reactions, leading to improved electrochemical performance. Numerous strategies for SEI construction on Zn anodes have been investigated. Key approaches include:

1. **Electrolyte Engineering**:^[^ ^3^ ^]^ This approach modifies the electrolyte composition to manipulate the SEI formation process and enhance its stability. Common strategies include the introduction of Inorganic additives: Salts such as ZnF_2_ and LiTFSI are commonly used to promote the formation of inorganic‐rich SEIs, enhancing their chemical stability. Organic additives: Molecules with specific functional groups (e.g., ─COOH, ─NH_2_) can facilitate the development of organic–inorganic composite SEIs with improved ionic conductivity and flexibility. Water‐in‐salt electrolytes: These concentrated electrolytes suppress water activity and promote the formation of denser, more robust, and inorganic‐rich SEIs, significantly improving anode stability.
2. **Surface Coating Modification**:^[^ ^4^ ^]^ Directly coating the Zn anode surface with protective layers is another effective method. Commonly used coatings include, for example, carbon‐based materials:^[^ ^5^ ^]^ Graphene, carbon nanotubes, and porous carbon structures exhibit high conductivity and large surface areas, helping to alleviate volume changes during cycling and mitigate dendrite formation. Metal oxides:^[^ ^6^ ^]^ Metal oxides, like ZnO and TiO_2_, possess high chemical stability in aqueous electrolytes and provide a physical barrier to reduce corrosion and regulate Zn deposition for a more homogenous and dendrite‐free surface.
3. **Artificial SEI Layers**: Pre‐forming artificial SEI layers with defined properties is another strategy to directly control the SEI's functionality and ensure optimal protection of the Zn anode. Techniques such as: atomic layer deposition^[^ ^7^ ^]^ and chemical vapor deposition: Enable the precise deposition of uniform, conformal layers of materials like metal oxides, sulfides, and even carbon‐based materials, providing a robust protective layer that promotes controlled Zn nucleation and minimizes dendrite growth.
4. **Structural Engineering of Zn Anodes**:^[^ ^8^ ^]^ Tailoring the anode's architecture and morphology is an additional strategy to increase the effective surface area, reduce local current density, and achieve more uniform Zn deposition. Common structural modifications include 3D porous structures: Employing porous Zn anodes, such as foams and meshes, helps accommodate the volume expansion and contraction during cycling and distributes the zinc ion flux, preventing the emergence of localized hot spots for dendrite formation. Surface texturing: Introducing controlled surface features like micro‐patterns or grooves on the Zn anode can help direct the Zn deposition toward specific sites, ensuring homogenous growth and minimizing the likelihood of dendrite nucleation.

While these methods offer promising avenues for enhancing zinc anode stability, many approaches focus primarily on physical barriers or uniformly modifying the surface. However, a key challenge lies in achieving atomic‐level control over the zinc deposition process and simultaneous suppression of parasitic reactions through SEI engineering. A functional SEI should not only act as a passive physical barrier, but it should actively participate in ion transport, regulating Zn ion flux while suppressing undesired reactions with the electrolyte. Our study tackles this challenge by developing a unique approach to Zn anode stabilization based on the formation of a robust, functional SEI. By introducing a ZnSiF_6_ additive to the ZnSO_4_ electrolyte, we were able to successfully induce the formation of a stable and unique fluorine‐rich SEI on the Zn anode surface. This SEI, primarily composed of a porous framework of alkali zinc sulfate (ZHS) infused with ZnF_2_, exhibits a hierarchical structure, creating nanometer‐sized channels that enhance zinc ion transport within the SEI layer. This unique structure allows for a remarkable synergy between rapid desolvation of Zn^2+^ ions, high ionic conductivity, effective passivation against corrosion, and significantly reduced hydrogen evolution.

Through comprehensive experimental and theoretical investigations, we elucidated the formation mechanism and functionality of the ZnSiF_6_‐derived SEI. Density functional theory (DFT) calculations and analyses of the Zn^2+^‐solvated structure provided compelling evidence for the decomposition of SiF_6_^2−^ ions as the primary driving force for SEI formation, resulting in a ZnF_2_‐enriched interphase. To delve into the microscopic structural details and understand the SEI formation process, we employed advanced characterization techniques including focused ion beam (FIB) milling combined with spherical aberration‐corrected transmission electron microscopy (Cs‐corrected TEM), X‐ray photoelectron spectroscopy (XPS), and Time‐of‐Flight Secondary Ion Mass Spectrometry (ToF‐SIMS). Our findings reveal the presence of a hierarchical SEI with a unique distribution of ZHS and ZnF_2_, confirming the successful modification of the interface and the key role played by the ZnSiF_6_ additive. These insights were further corroborated by electrochemical studies in Zn||Zn symmetric and Zn||Cu asymmetric cell configurations, demonstrating impressive cycling stability exceeding 3000 h at 0.5 mA cm^−2^ (and 0.5 mAh cm^−2^), exceeding 560 h at 10 mA cm^−2^ (and 10 mAh cm^−2^). Our strategy enabled remarkably high Zn utilization and offered a comprehensive picture of how the carefully engineered SEI contributes to stable and high‐performance aqueous ZIBs.

## 2 Results and Discussion

### 2.1 Regulation of Zn^2+^ Solvation Structure by ZnSiF_6_

**Figure 1:**
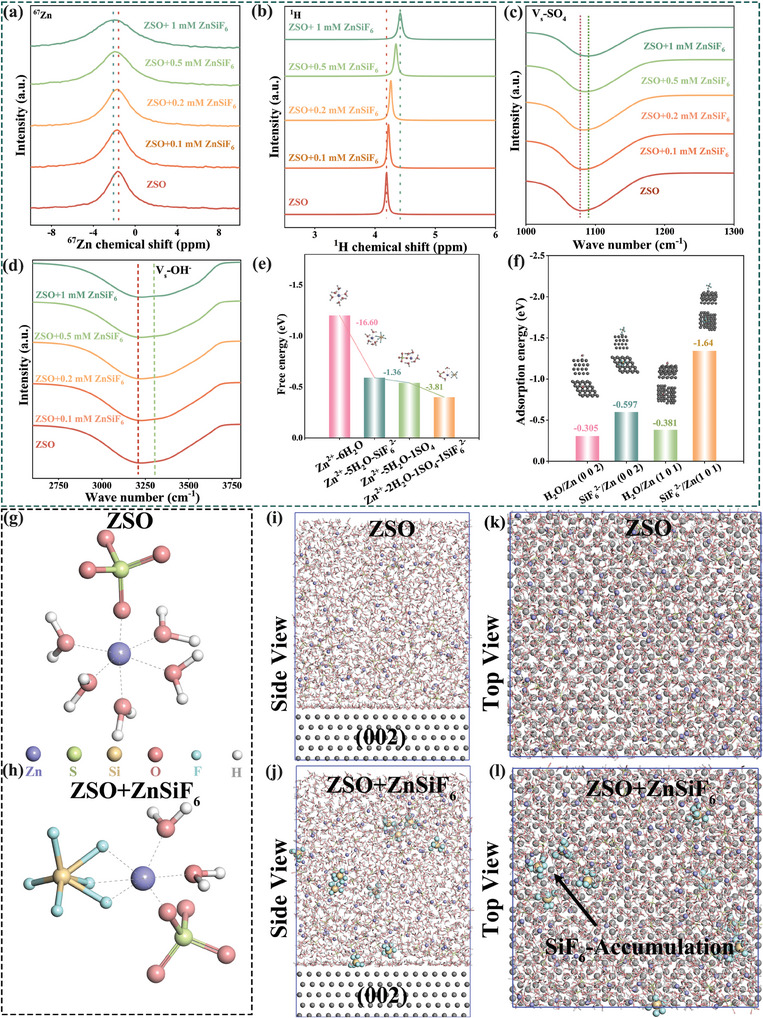
Regulation of the solvation sheath of Zn^2+^ by the addition of ZnSiF_6_. a) ^67^Zn NMR; b) ^1^H NMR and FT‐IR spectra c) SO_4_^2−^ d) OH^−^ of ZSO, ZSO+0.1 mm ZnSiF_6_, ZSO+0.2 mm ZnSiF_6_, ZSO+0.5 mm ZnSiF_6_ and ZSO+1 mm ZnSiF_6_; e) Free energies of Zn^2+^−6H_2_O, Zn^2+^−5H_2_O‐1SiF_6_^2−^, Zn^2+^−5H_2_O‐1SO_4_^2−^, and Zn^2+^−2H_2_O‐1SO_4_^2−^−1SiF_6_^2−^ and corresponding structures; f) Adsorption energies of H_2_O and 1SiF_6_^2−^ molecules on Zn(002) and(101) facets respectively; g,h) partially enlarged snapshots of the solvation structure of Zn^2+^; i–l) Side view and Top view of Zn(002) charged to 0.1 e per atom in ZSO, ZSO+ZnSiF_6_ electrolyte, respectively.

To understand the influence of the ZnSiF_6_ additive on the Zn^2+^ solvation structure within the electrolyte, we performed nuclear magnetic resonance (NMR) and Fourier‐transform infrared (FT‐IR) spectroscopic studies. NMR spectroscopy revealed that as the concentration of ZnSiF_6_ in 2 mol L^−1^ (M) ZnSO_4_ increases from 0 to 1 mm (marked as ZSO, ZSO+0.1 mm ZnSiF_6_, ZSO+0.2 mm ZnSiF_6_, ZSO+0.5 mm ZnSiF_6_, and ZSO+1 mm ZnSiF_6_, respectively), the ^67^Zn resonance peak gradually shifts downfield by 1 ppm (**Figure** **1**a). This indicates that the solvation shell around the Zn^2+^ ions is changing, specifically through the displacement of water molecules and coordination with the added SiF_6_^2−^ anions. The broadening of the ^67^Zn peaks with increasing ZnSiF_6_ further supports the formation of Zn^2+^‐SiF_6_^2−^ complexes.^[^ ^9^ ^]^ Conversely, ^1^H NMR analysis (Figure 1b) displayed the opposite trend‐peak narrowing with increasing ZnSiF_6_, likely due to the displacement of water molecules from the primary solvation sphere of the Zn^2+^ by the additive anions. The displaced water then becomes free to form hydrogen bonds with F atoms present in the SiF_6_^2−^ anions, resulting in less defined hydrogen bonding configurations for these water molecules.

Further support for these structural changes was obtained through FT‐IR and Raman spectroscopic studies of SO_4_^2−^ and OH^−^ vibrational modes (Figure 1c; Figures S1–S3, Supporting Information). The FT‐IR spectra showed that the SO_4_^2−^ stretching vibration (Vs‐SO_4_^2−^) shifts from 1078 to 1089 cm^−1^ with increasing ZnSiF_6_ concentration (Figure 1c), indicating strengthening of the S─O bond in the sulfate anion. This suggests that the addition of ZnSiF_6_ directly affects the interaction between Zn^2+^ and SO_4_^2−^. Figure 1d depicts the changes observed in O─H stretching vibrations (Vs─OH^−^) within the range of 3000–4000 cm^−1^, corresponding to strong and medium hydrogen bond configurations in water molecules. Upon introducing ZnSiF_6_, a clear attenuation of the strong H─bonds and a concurrent intensification of medium H─bonds were observed. These modifications to the hydrogen bonding network suggest a weaker water structure, consistent with a more facile desolvation process, likely related to interactions between H and F atoms. This conclusion is further supported by DFT calculations examining the free energies of various Zn^2+^ solvation configurations. Figure 1e (and Figure S4, Supporting Information) clearly shows that a Zn^2+^−2H_2_O‐SiF_6_^2−^‐SO_4_^2−^ configuration is the most energetically favorable solvation structure in the ZSO+ZnSiF_6_ electrolyte.

To understand how the ZnSiF_6_ additive might impact the electrochemical interface, we employed DFT calculations to evaluate the adsorption energies of key solution components on different Zn crystal facets ((002) and (101)). Figure 1f (and Figure S5, Supporting Information) clearly show that, unlike water molecules that interact weakly with Zn, SiF_6_^2−^ exhibits strong adsorption onto the Zn surface. This suggests a strong affinity of SiF_6_^2−^ anions toward the Zn surface, indicating potential interactions and the formation of a stable interface. We explored these interactions using molecular dynamics (MD) simulations (Figure 1g,h; Figure S6–S8, Supporting Information) and observed that in the base ZSO electrolyte, Zn^2+^ is primarily coordinated with 5 H_2_O molecules and a single SO_4_^2−^ anion. However, in the ZSO+ZnSiF_6_ electrolyte, SiF_6_^2−^ anions actively participate in the Zn^2+^ solvation shell, replacing one water molecule, supporting our spectroscopic observations.

Further investigation of the interface using MD simulations under charging conditions (with an applied charge of 0.1 e Zn^−1^ atom) is presented in Figure 1i,l (and Figures S9–S11, Supporting Information). In the modified ZSO+ZnSiF_6_ electrolyte, a clear accumulation of SiF_6_^2−^ anions are observed on the Zn(002) electrode surface, a trend absent in the standard ZSO electrolyte simulations. This preference for accumulating near the electrode suggests that SiF_6_^2−^ undergoes electrochemical decomposition during charging to form the stable SEI. To investigate the solution stability, we tested different ZnSiF_6_ concentrations in ZSO. Our results indicate that precipitate formation was observed at concentrations higher than 1 mm ZnSiF_6_, but only negligible pH changes occurred below 0.5 mm ZnSiF_6_. (Figures S12 and S13, Supporting Information). Therefore, for our detailed investigation and clear comparison, we selected ZSO as the baseline and a 2 m ZnSO_4_ electrolyte containing 0.5 mm ZnSiF_6_ (ZSO‐ZnSiF_6_) as the modified electrolyte.

The NMR, FT‐IR, and DFT results collectively demonstrate that the ZnSiF_6_ additive effectively alters the solvation structure of Zn^2+^ ions in the electrolyte, replacing water molecules in the primary solvation shell. This modification promotes desolvation, weakens the hydrogen bonding network in water, and increases the interaction between Zn^2+^ and SO_4_^2−^, potentially contributing to enhanced electrochemical performance.

### 2.2 Corrosion Inhibitions

**Figure 2:**
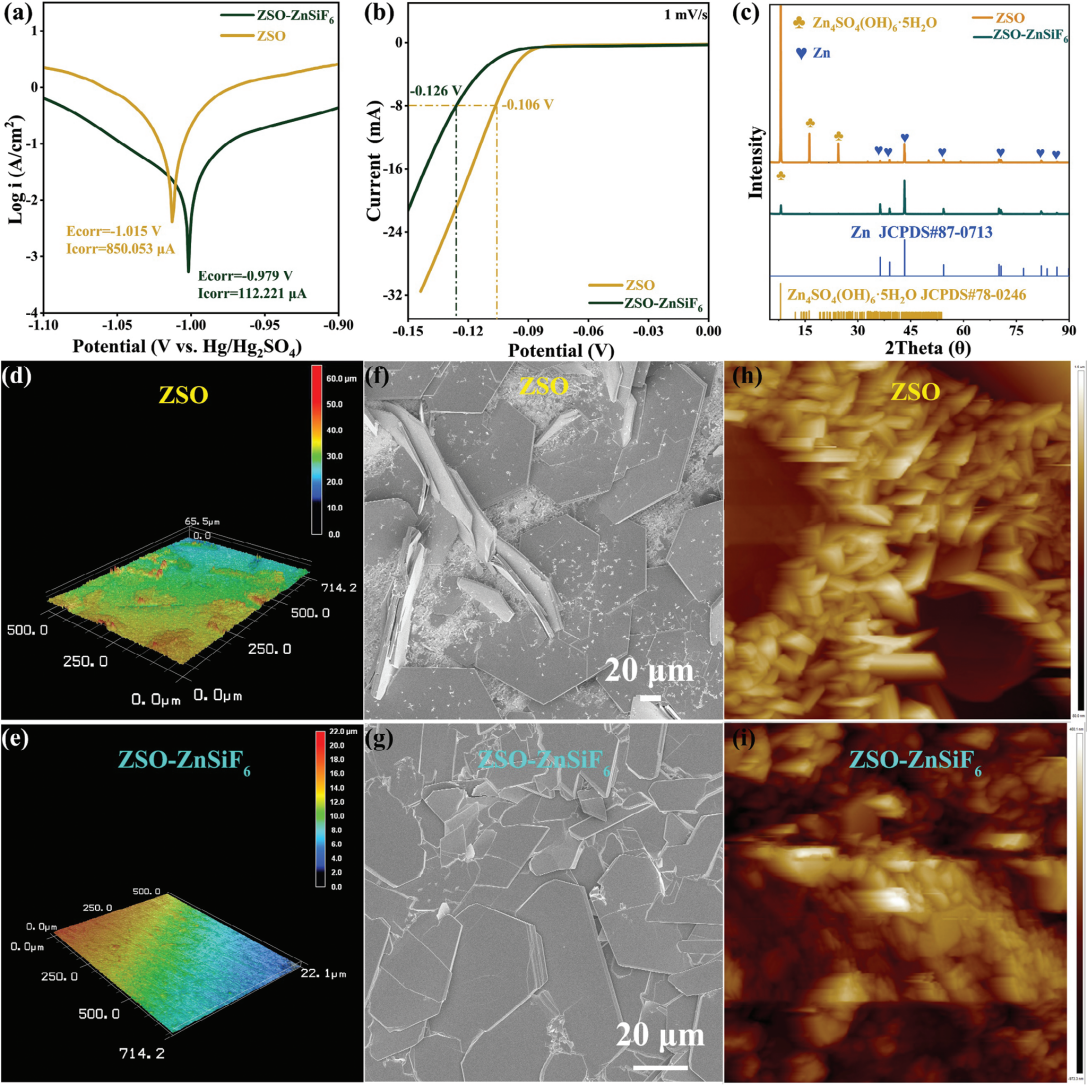
Theoretical and experimental investigations into the inhibition of the side reactions on the Zn anode. a) Tafel slope curves with a three‐electrode configuration in ZSO and ZSO‐ZnSiF_6_ electrolytes; b) Corresponding LSV curves; c) XRD patterns of Zn anodes soaked in electrolytes for 20 days. d–i) CLSM, SEM, and AFM images (denoised by NanoScope Analysis Software) of Zn anodes soaked in ZSO and ZSO‐ZnSiF_6_ electrolytes for 20 days respectively.

Electrochemical measurements revealed the remarkable impact of the ZnSiF_6_ additive in enhancing the corrosion resistance of the Zn anode. As shown in the Tafel polarization curves (**Figure** **2**a; Figure S14, Supporting Information), the Zn electrode immersed in the ZSO electrolyte displayed a significant corrosion current density of 850.05 µA cm^−2^ and a corrosion potential of −1.015 V (vs Hg/HgSO_4_). However, in the ZSO‐ZnSiF_6_ electrolyte, the corrosion current density plummeted to 112.22 µA cm^−2^, and the corrosion potential shifted slightly more positively to −0.979 V. This strongly indicates that the presence of SiF_6_^2−^ anions significantly inhibits the corrosion process. These observations are corroborated by Linear Sweep Voltammetry (LSV) data (Figure 2b; Figure S15, Supporting Information), where Zn||Ti cells in the ZnSiF_6_‐modified electrolyte displayed an expanded potential window for both hydrogen and oxygen evolution, indicative of reduced water decomposition. These findings align with the Tafel polarization data and highlight the role of the additive in suppressing undesirable electrochemical reactions at the anode/electrolyte interface. To further investigate the long‐term impact of the ZnSiF_6_ additive, we immersed Zn anodes in the respective electrolytes (ZSO and ZSO‐ZnSiF_6_) for 20 days and characterized the surface morphology and composition using XRD, SEM‐mapping, and Energy‐dispersive X‐ray Spectroscopy (EDS). As shown in Figure 2c–i, the anode soaked in ZSO exhibited substantial accumulation of Zn_4_SO_4_(OH)_6_·5H_2_O (ZHS) byproducts, which act as insulators and hinder ion transport. In contrast, the ZnSiF_6_‐modified electrolyte yielded significantly less byproduct formation, underscoring the ability of the additive‐induced SEI to suppress undesired side reactions, leading to a more stable interface. Additional insights were gleaned through high‐resolution imaging. Confocal laser scanning microscopy (CLSM) revealed substantial surface roughening on the anode soaked in the ZSO electrolyte. As seen in Figure 2d, the anode developed numerous micrometer‐scale protrusions, suggesting uneven Zn dissolution and surface damage. These features were also apparent in SEM images (Figure 2f), revealing extensive surface defects and the formation of irregular flakes, indicative of corrosion. In striking contrast, the Zn anode immersed in ZSO‐ZnSiF_6_ exhibited a significantly smoother and denser surface (Figure 2e,g), showcasing enhanced corrosion resistance due to the protective nature of the additive‐induced SEI. Atomic Force Microscopy (AFM) was used to quantitatively characterize the anode surface roughness before and after cycling. Figure 2h,i presents the 3D AFM topography images, clearly showcasing the reduction in surface roughness upon using the ZnSiF_6_ additive. Further quantitative analysis (Figure 2j,k) showed that Zn anodes cycled in ZSO‐ZnSiF_6_ retained a smoother surface, with an average surface roughness of 460.1 nm compared to 1500 nm for anodes cycled in the ZSO electrolyte. The results from long‐term immersion tests, coupled with high‐resolution microscopy characterization, confirm the remarkable role played by the ZnSiF_6_ additive in creating a robust SEI that hinders corrosion, suppresses undesirable by‐product formation and promotes a more compact and homogeneous Zn anode morphology.

### 2.3 Performance of Symmetric or Half Cells

**Figure 3:**
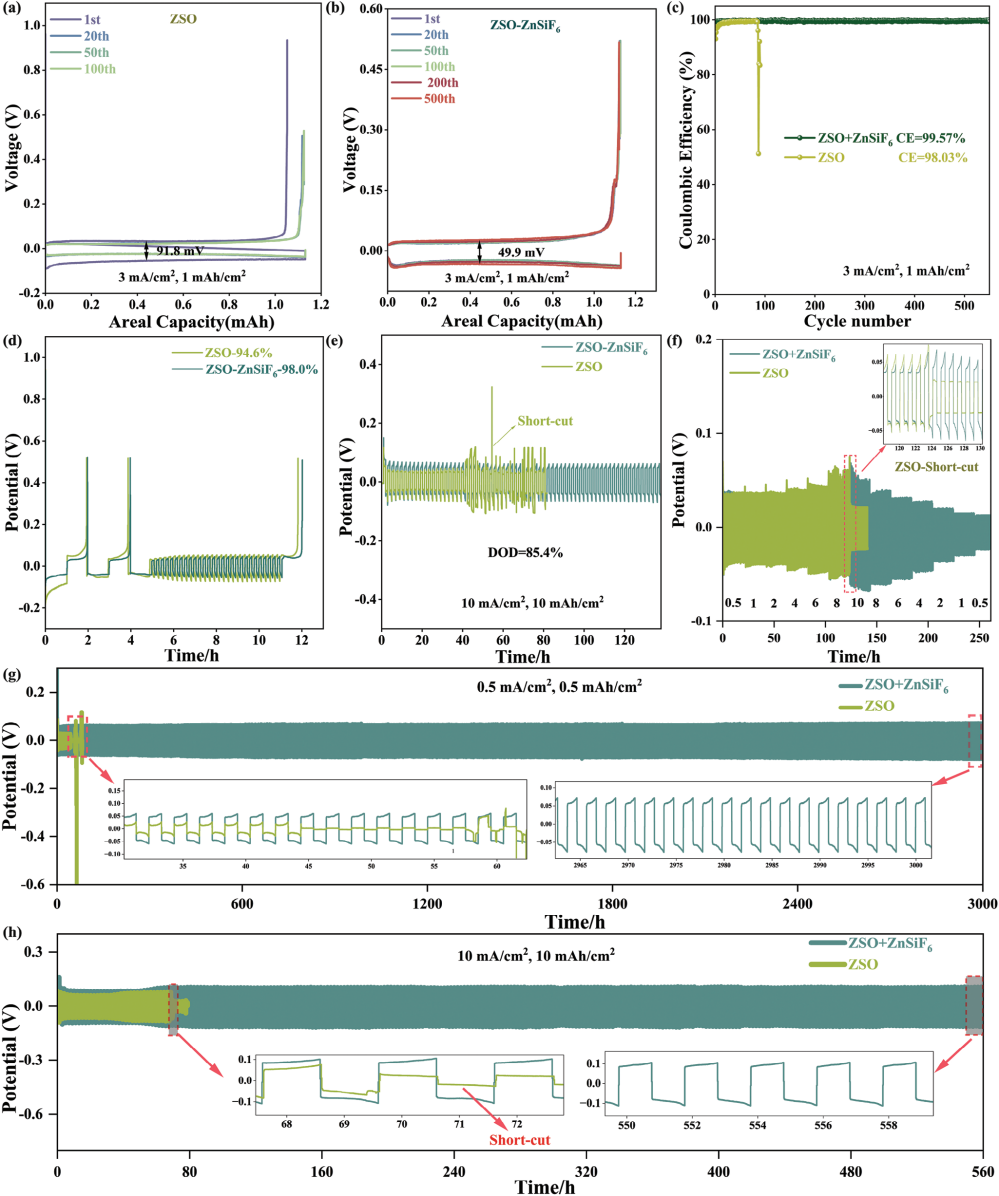
Electrochemical performance employing ZSO and ZSO‐ZnSiF_6_ as electrolytes. Voltage profiles of Zn||Cu cells in a) ZSO and b) ZSO‐ZnSiF_6_ electrolytes for various cycles. c) Coulombic efficiencies of Zn||Cu cells at 3 mA cm^−2^ and 1 mAh cm^−2^; d) Reservoir protocol of Zn||Cu half cells. e) DOD of Zn anodes in Zn||Zn cells; f) Rate performance of Zn||Zn symmetric cells; g,h) Long‐term galvanostatic cycling performance of Zn||Zn symmetric cells at 0.5 and 10 mAh cm^−2^.

We further assessed the impact of the ZnSiF_6_ additive on electrochemical performance using both Zn||Zn symmetric and Zn||Cu asymmetric cells. As shown in **Figure** **3**a,b, galvanostatic cycling tests at 3 mA cm^−2^ and 1 mAh cm^−2^ areal capacity in Zn||Cu cells revealed drastically improved stability and coulombic efficiency (CE) for cells containing ZnSiF_6_ in the electrolyte. While the cell using the base ZSO electrolyte (Figure 3a,c) demonstrated fluctuating CE and greater polarization of 91.8 mV ultimately failed after 100 cycles, the ZSO‐ZnSiF_6_ cell maintained a stable voltage profile and smaller polarization of 49.9 mV with an average CE of 99.57% for over 600 cycles (Figure 3b,c). Figure 3c showcases the drastic difference in capacity retention between these systems, further highlighting the positive effects of the ZnSiF_6_ additive in mitigating the formation of “dead zinc” and facilitating reversible Zn plating and stripping. To gain a more precise understanding of Zn utilization, a “reservoir” protocol was applied to Zn||Cu cells (Figure 3d). In these experiments, the ZSO‐ZnSiF_6_ cell demonstrated an impressive 98.0% average CE, with stable and consistent cycling performance. The ZSO cell, on the other hand, displayed fluctuating voltages and ultimately failed to complete the protocol, further highlighting its susceptibility to parasitic side reactions. The impact of ZnSiF_6_ on Zn utilization was further investigated by evaluating the Depth of Discharge (DOD) in symmetric Zn||Zn cells under various cycling conditions. The results, summarized in Figure 3e, show that cells utilizing the ZnSiF_6_ additive enabled a remarkable 85.4% DOD while maintaining excellent stability across a range of current densities. In contrast, Zn||Zn cells using the ZSO electrolyte suffered from severe fluctuations in cycling profiles and exhibited premature failure. These results highlight a critical advantage offered by ZnSiF_6_ – increased reversible zinc utilization by minimizing parasitic reactions and improving anode reversibility, key for achieving long‐lasting and efficient ZIBs. This enhanced stability and reversibility were further validated in Zn||Zn symmetric cells tested under various current density conditions (0.5 to 10 mA cm^−2^). Figure 3f demonstrates excellent rate capability for the cell employing the modified ZSO‐ZnSiF_6_ electrolyte, signifying rapid charge transfer at the electrode interface. The cell employing ZSO‐ZnSiF_6_ achieved a record‐breaking 3000‐h cycling lifespan at 0.5 mA cm^−2^ with an areal capacity of 0.5 mAh cm^−2^. The exceptional cycle life obtained in this test surpasses any reported value in recent literature^[^ ^10^ ^]^ using similar electrolytes or electrolyte additives (Figure 3g; Figure S16, Supporting Information). Even at a higher current density of 10 mA cm^−2^ and an areal capacity of 10 mAh cm^−2^, this cell exhibited impressive cycling stability exceeding 560 h. In stark contrast, the symmetric cell employing the standard ZSO electrolyte showed considerably poor cycling stability, failing within just 50 h (Figure 3g) and only 58 h at the respective current densities (Figure 3h).

These remarkable enhancements in both symmetric and asymmetric cells unequivocally establish the exceptional stabilizing effect of the ZnSiF_6_ additive on zinc anodes. The high Coulombic efficiency, extended cycle life, and improved rate capability, coupled with the analysis of byproduct formation, directly support the crucial role of the robust and well‐defined SEI facilitated by ZnSiF_6_. This optimized interphase suppresses parasitic reactions, and promotes homogeneous Zn deposition and dissolution, leading to ultra‐stable Zn anodes, exceeding the performance of similar ZIB systems reported previously (Figure S16, Supporting Information).

### 2.4 Zn Deposition/Dissolution Kinetics and Nucleation Behavior

**Figure 4:**
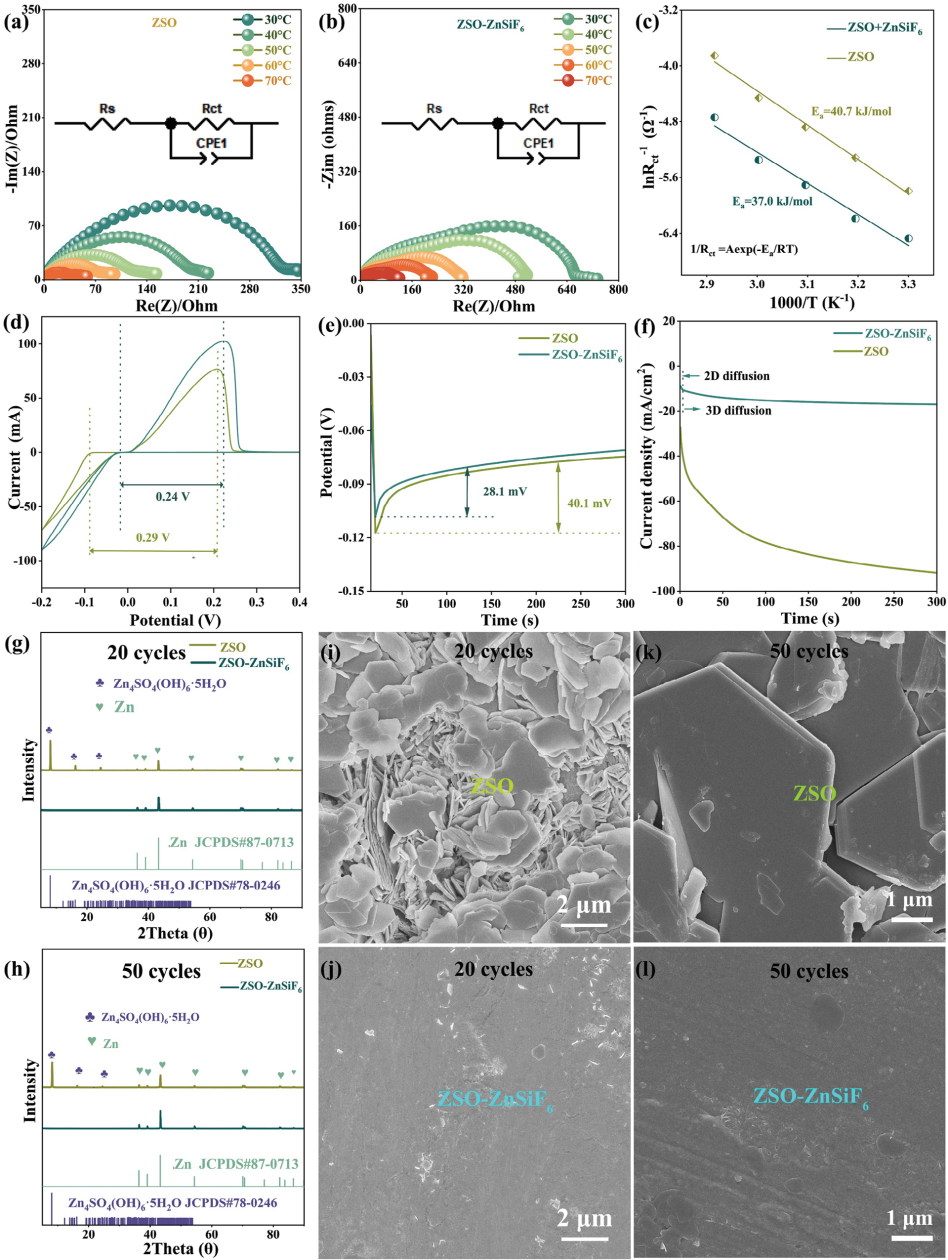
Dynamic kinetics in ZSO and ZSO‐ZnSiF_6_ electrolytes and morphology of Zn anodes after different cycles. a–c) Nyquist plots of Zn electrodes and activation energies; d) CV measurements of Zn||Ti half cells; e) Nucleation overpotential of Zn||Ti asymmetric cells; f) CA of Zn||Zn cells at a −150 mV overpotential; g,h) XRD patterns; i–l) SEM images of the Zn anode in different electrolytes after 20 and 50 cycles.

To understand the origin of enhanced electrochemical performance, we conducted a systematic investigation of Zn deposition/dissolution kinetics and nucleation behavior in both electrolytes using various electrochemical techniques. To gain quantitative insight into the kinetics, we determined the activation energies for the charge transfer process. This was achieved through temperature‐dependent EIS measurements and analysis of the obtained charge‐transfer resistance (Rct) values (Tables S1–S2, Supporting Information). **Figure** **4**a,b shows the Nyquist plots obtained for both electrolytes over a range of temperatures (30–70 °C). We observe a decrease in Rct with increasing temperature, consistent with faster kinetics. To quantify the effect, we applied the Arrhenius equation 1/ R_ct_ =  Aexp(− E_a_/RT), where E_a_ is the activation energy, R is the ideal gas constant, and T is the absolute temperature. The Arrhenius plots based on the Rct values obtained are presented in Figure 4c. Linear fitting of this data allows us to extract the activation energy (E_a_) values, revealing a clear distinction between the two electrolytes. In the ZnSiF_6_‐modified electrolyte, the calculated E_a_ value is 37.0 kJ mol^−1^, significantly lower than the 40.7 kJ mol^−1^ obtained for the ZSO electrolyte. This finding strongly suggests that ZnSiF_6_ promotes faster charge transfer kinetics, possibly facilitating desolvation and improving ionic mobility at the electrode interface. To understand the impact on Zn nucleation, we performed cyclic voltammetry (CV) on Zn||Ti asymmetric cells, as shown in Figure 4d. The CV profiles show distinct differences, where the cell utilizing ZSO‐ZnSiF_6_ exhibited a narrower separation between the anodic and cathodic peaks, suggesting improved reversibility for the Zn deposition and dissolution process. This implies smoother and more facile Zn nucleation on the electrode. We further confirmed these observations through nucleation overpotential (NOP) measurements. Figure 4e highlights that the Zn plating/stripping overpotential for the ZSO‐ZnSiF_6_ electrolyte (28.1 mV) is substantially lower than the ZSO electrolyte (40.1 mV). This decrease in NOP suggests a significant reduction in the energy barrier for Zn nucleation. These results point toward a unique mechanism of SEI‐mediated Zn deposition, promoted by the ZnSiF_6_ additive. Chronoamperometry (CA) measurements on Zn||Zn symmetric cells provided further insights into the impact of the modified electrolyte on Zn deposition kinetics and behavior. Figure 4f displays the CA profiles for both electrolytes at an applied overpotential of −150 mV. In the standard ZSO electrolyte, a rapid increase in the absolute current density was observed, lasting for ≈60 s. This initial response is typical for 2D diffusion‐controlled processes, indicative of a fast increase in active surface area, which usually leads to porous and inhomogeneous zinc deposition and facilitates dendrite growth. The ZnSiF_6_‐modified electrolyte displayed a remarkably different CA profile, with a smooth, steadily decreasing current response throughout the 300‐s measurement period. This profile strongly suggests a 3D diffusion‐controlled deposition process, indicating uniform and controlled Zn nucleation and growth, which aligns with the previous observations of smooth morphology and improved stability. The CA results, coupled with the reduced NOP in the ZnSiF_6_‐containing system, point toward an SEI‐mediated modification of Zn nucleation and deposition mechanisms, enabling stable, homogenous plating that significantly contributes to enhanced electrochemical performance. The impact of ZnSiF_6_ on the crystalline structure of the plated zinc was first analyzed via XRD after 20 and 50 cycles (Figure 4g,h). Notably, Zn_4_SO_4_(OH)_6_·5H_2_O, a known byproduct of parasitic reactions often contributing to performance decline, was prominent in the ZSO cell but minimal in the cell employing the ZnSiF_6_‐modified electrolyte. The preferred deposition of zinc along the (002) crystal plane in the ZSO‐ZnSiF_6_ system is also reflected by the higher (002)/(101) peak intensity ratios after both 20 and 50 cycles (Figure S17, Supporting Information), further suggesting altered deposition kinetics influenced by the SEI. These findings reinforce the efficacy of the additive‐induced SEI in suppressing unwanted side reactions, further supported by the surface morphologies observed through SEM (Figure 4i–l; Figures S18–S21, Supporting Information). In the ZSO electrolyte, Zn plating led to loosely‐packed, vertically grown structures after 20 cycles, evolving to densely packed, larger hexagonal formations by the 50th cycle (Figure 4i,k), typical of an accelerated, uneven 2D deposition mode prone to dendrite growth. Conversely, in the presence of ZnSiF_6_, SEM revealed a considerably smoother and more compact morphology. The Zn deposited as horizontally stacked layers after both 20 and 50 cycles (Figure 4j,l). These distinct morphologies correlate with the two distinct deposition modes observed through chronoamperometry and confirm that ZnSiF_6_ significantly alters the Zn deposition mechanism toward a 3D mode, contributing to improved anode stability and electrochemical performance.

**Figure 5:**
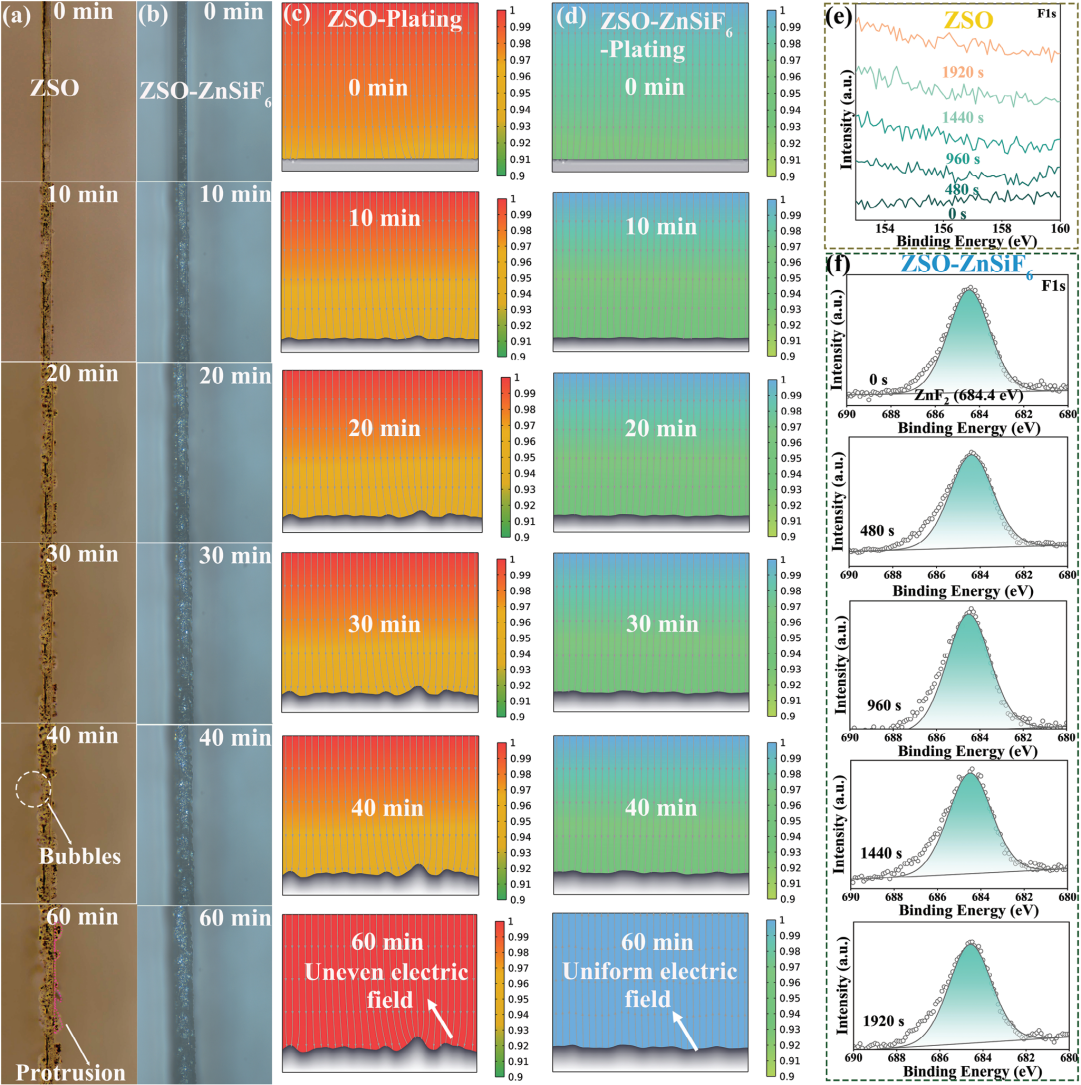
Structural and compositional characterization of Zn anodes. a,b) In situ optical images at 5 mA cm^−2^; c,d) COMSOL simulation of Zn corrosion and HER during Zn plating/stripping process; and e,f) In‐depth XPS spectra of F 1s on the Zn anodes after 20 cycles in ZSO and ZSO‐ZnSiF_6_.

In situ optical microscopy studies further confirmed these distinct deposition behaviors, visually capturing the dynamic plating processes for both electrolytes. In the ZSO electrolyte, the plating was highly irregular, and rapid growth of dendrites and hydrogen bubbles was observed at the anode surface within just 60 min at a current density of 5 mA cm^−2^ (**Figure** **5**a). In stark contrast, the ZSO‐ZnSiF_6_ electrolyte showed a remarkably homogeneous and stable deposition process with uniform zinc plating and minimal H_2_ bubble evolution during the entire duration of the experiment (Figure 5b). This visual confirmation directly highlights the impact of the modified SEI in suppressing parasitic reactions and facilitating uniform Zn plating. To explore how this morphological contrast is tied to the local electric field and deposition behavior, we employed finite element modeling (FEM) simulations using COMSOL Multiphysics. As shown in Figure 5c,d and Figures S22–S23 (Supporting Information), in the ZSO electrolyte, large potential gradients formed on the electrode surface, indicative of a highly localized current distribution that drives preferential Zn plating at specific high‐potential zones (marked by arrows). This inherently non‐uniform plating readily leads to dendritic growth and performance degradation. Conversely, the simulation with the ZnSiF_6_‐modified electrolyte showcases a significantly more homogenous potential distribution throughout the entire Zn plating process, confirming a more even current flow and deposition, corroborating the uniform Zn morphology observed experimentally. This demonstrates the critical role of the ZnSiF_6_‐induced SEI in stabilizing the local electric field during plating, thus minimizing local hotspots and preventing detrimental dendrite formation.

### 2.5 ZnSiF_6_‐Derived SEI Characterization

To understand the SEI structure, composition, and formation mechanism in greater detail, we employed a multi‐faceted characterization approach encompassing XPS, FIB milling combined with high‐resolution Scanning Transmission Electron Microscopy (STEM), and ToF‐SIMS.

To definitively confirm the involvement of the ZnSiF_6_ additive in SEI formation, we analyzed the composition of the anode surface using XPS combined with Ar^+^ sputtering to obtain depth profile information (Figure 5e,f; Figure S24, Supporting Information). While no F signal was observed on the surface of the anode cycled in the base ZSO electrolyte, confirming the absence of fluorine‐containing species, the ZnSiF_6_‐treated cell exhibited a distinct peak in the F 1s spectra corresponding to Zn─F bonds (Figure 5f). This peak, observed at a binding energy of 684.4 eV, arises from ZnF_2_, confirming the electrochemical decomposition of the SiF_6_^2−^ anions and incorporation of F into the SEI. Importantly, the persistent F signal, even after extensive sputtering, confirms the formation of a stable fluorine‐rich SEI layer of a certain thickness on the anode, validating our proposed mechanism of SEI formation driven by ZnSiF_6_ decomposition.

**Figure 6:**
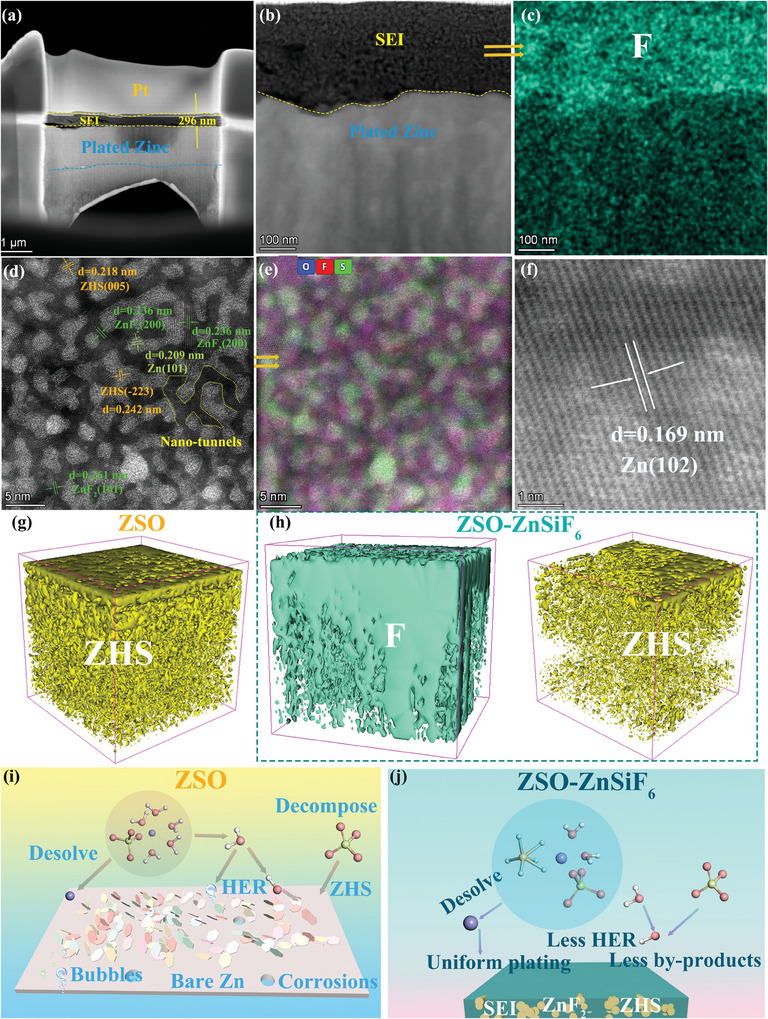
Composition and microstructure of SEI on cycled Zn anodes. a) FIB images; b–f) high‐angle annular dark field (HAAD images and mappings; g,h) TOF‐SIMS 3D rendered images after 20 cycles; i,j) Schematic illustrations of the underlying mechanisms in the ZSO and ZSO‐ZnSiF_6_ electrolytes.

To further understand the nanoscale structure of the SEI and confirm the presence of a distinct fluorine‐rich layer, we performed a comprehensive high‐resolution characterization using FIB milling to create precise cross‐sections of the Zn anodes, which were subsequently imaged and analyzed by STEM coupled with energy‐dispersive X‐ray spectroscopy (EDX). **Figure** **6**a shows the cross‐sectional STEM image of a Zn anode cycled in the ZSO‐ZnSiF_6_ electrolyte for 20 cycles. A well‐defined layer on the Zn surface is evident (for additional images, refer to Figures S25–S27, Supporting Information), confirming the successful formation of an SEI. A closer examination of the SEI reveals a distinct bi‐layer morphology (Figure 6b), composed **of Region I**: The outer layer, characterized by a unique hierarchical morphology of ≈296 nm in thickness, containing both crystalline and amorphous components. **Region II: An** inner layer of uniformly deposited Zn metal directly contacting the bulk Zn anode. EDX analysis within Region I (Figure 6c) showcases a homogeneous distribution of fluorine across this layer, suggesting a fluorine‐rich interphase, consistent with our XPS observations. Further high‐resolution STEM analysis of Region I (Figure 6d) provides more structural details, clearly showcasing that porous ZHS forms the foundational skeletal framework for the SEI. The ZHS framework contains well‐defined inter‐particle voids, and we observed nano‐sized crystalline domains within this structure exhibiting lattice spacings that directly correspond to ZnF_2_ (Figure 6d). This confirms the successful integration of ZnF_2_ within the porous ZHS, forming interconnected channels for efficient zinc ion transport, a hallmark of our hierarchical SEI design. This composite architecture is visualized more directly in the HAADF‐STEM image (Figure 6e), along with the elemental mapping, which further confirms the presence of both ZnF_2_ and ZHS. The uniform distribution of oxygen and sulfur, which correspond to the components of ZHS, alongside the localized signal for F originating from the embedded ZnF_2_, confirms our proposed composite SEI structure. Moving inward, Region II exhibits well‐ordered Zn atomic planes, indicating the uniformly plated Zn metal directly interfacing with the SEI (Figure 6f).

To provide a more comprehensive visualization of the spatial distribution of different components within the SEI layer, we employed ToF‐SIMS. Figure 6g,h and Figure S28 (Supporting Information) show 3D renderings based on ToF‐SIMS data for both ZSO and ZSO‐ZnSiF_6_ electrolytes. In the ZSO‐treated sample, ZnS dominates the near‐surface region alongside oxygen signals associated with the presence of Zn_4_SO_4_(OH)_6_·5H_2_O (ZHS), (Figure 6g). In contrast, the ZSO‐ZnSiF_6_‐treated Zn anode shows an intricate interweaving of F signals within the ZHS framework (Figure 6h), providing compelling visual evidence of a multi‐component, fluorine‐rich SEI, distinctly different from that obtained without the additive. Figure S29 (Supporting Information) presents the depth profiles of various elemental signals, which provide further supporting evidence. We observe the persistence of a strong F signal throughout the analyzed depth, indicating the uniform presence of fluorine across the entire SEI thickness, corroborating our earlier observations from XPS. Notably, the signal for ZnFH_2_O, potentially arising from hydrated forms of ZnF_2_ within the SEI structure, suggests its presence both on the surface and within the SEI bulk. This heterogeneous distribution strongly points toward a hierarchical and multi‐component SEI design, unique to our system.

These meticulous characterizations, in conjunction with our DFT and MD analyses, allow us to present a comprehensive and visually compelling illustration of the mechanisms at play in both the base ZSO electrolyte and the modified electrolyte system (Figure 6i,j). Figure 6i showcases the detrimental scenario observed with the standard ZSO electrolyte. The accumulation of hydrated Zn^2+^ at the interface creates a highly competitive environment between Zn^2+^ deposition and water reduction, resulting in dominant H^+^ production, exacerbated corrosion of the Zn anode, and increased formation of insulating byproducts (predominantly ZHS). This further disrupts the uniformity of the local electric field at the anode surface, creating hot spots that accelerate non‐uniform Zn plating and facilitate the formation of harmful dendrites. These detrimental processes ultimately result in reduced Zn utilization and poor cycle life. Conversely, the introduction of ZnSiF_6_ significantly modifies the solvation environment and the subsequent electrochemical reactions at the anode, as highlighted in Figure 6j. The modified solvation shell around Zn^2+^, with partial replacement of water by SiF_6_^2−^ anions, coupled with the preferential decomposition of SiF_6_^2−^ on the Zn surface, leads to the formation of the unique, fluorine‐rich SEI discussed above. This hierarchically structured, robust SEI provides a robust barrier, effectively limiting water contact, and hindering HER and byproduct formation. Simultaneously, this SEI design promotes a smoother, more uniform distribution of Zn^2+^ ion flux. As a result, Zn nucleation becomes significantly more homogeneous, and the growth of zinc predominantly follows a 3D deposition mode, as corroborated by the electrochemical analysis and SEM characterizations. This 3D deposition effectively mitigates localized dendrite growth and results in compact, uniform Zn layers on the electrode surface.

### 2.6 Full Cell Performance and Pouch Cell Demonstration

**Figure 7:**
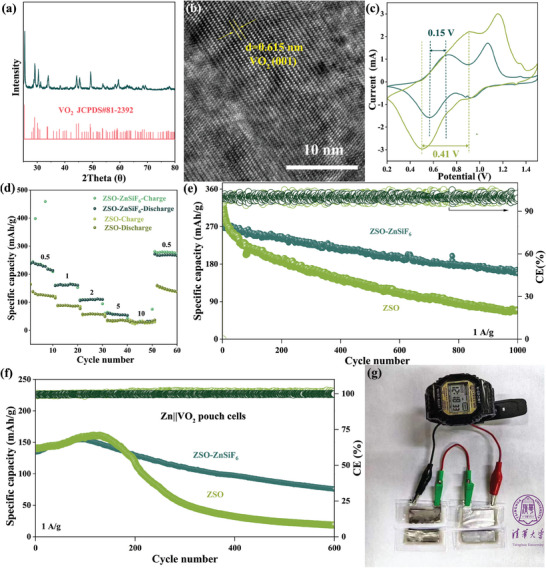
Characterization of VO_2_ and cycling performance of Zn||VO_2_ cells. a) XRD pattern of as‐prepared VO_2_; b) TEM image; c) CV measurement; d) Rate performance; e) Long‐term cycling performance at 1 A g^−1^; f) Cycling performance of Zn||VO_2_ pouch cells at 0.5 A g^−1^; g) Optical photograph of Zn||VO_2_ pouch cells.

Finally, to assess the efficacy of the ZnSiF_6_‐mediated SEI strategy in a practical setting, we tested the performance of full Zn‐ion battery cells using VO_2_ as the cathode material. The VO_2_ material was prepared via a hydrothermal synthesis method and its excellent crystallinity was confirmed by XRD and HRTEM (**Figure** **7**a,b, respectively). Figure 7c presents the CV data for the Zn||VO_2_ cells employing ZSO and ZSO‐ZnSiF_6_ electrolytes. Although the profiles exhibit characteristic redox peaks related to zinc insertion and de‐insertion in VO_2_, the cell with the modified electrolyte displays significantly lower peak separation between anodic and cathodic events, demonstrating faster Zn^2+^ diffusion and improved charge transfer kinetics enabled by the hierarchical SEI structure on the anode. Figure 7d demonstrates the excellent rate capability of Zn||VO_2_ full cells employing the ZnSiF_6_‐modified electrolyte, where consistently higher capacities are observed at every tested current density compared to those employing the ZSO electrolyte. When cycled at a constant current density of 1 A g^−1^, the benefits of ZnSiF_6_ became even more apparent (Figure 7e). The full cell with the ZnSiF_6_‐modified electrolyte achieved 70.4% capacity retention after 1000 cycles, exhibiting excellent long‐term stability. This is in stark contrast to the base ZSO electrolyte‐based cell, which demonstrated rapid degradation and maintained only 18.2% of its initial capacity after 1000 cycles.

These results showcase the direct benefits of implementing a stable, functional SEI for practical application in ZIBs. To further solidify this, we assembled Zn||VO_2_ pouch cells. The pouch cell constructed using the ZSO‐ZnSiF_6_ electrolyte (shown in Figure 7f,g) delivered a promising initial discharge capacity of 140 mAh g^−1^ at 0.5 A g^−1^ (Figure 7f), maintaining over 80% of this capacity even after 600 cycles, highlighting the practicality of our strategy. The overall enhancement in full cell performance, particularly the superior rate capability and exceptional cycle life achieved using the modified electrolyte, showcases the transformative effect of the engineered SEI in mitigating the challenges traditionally faced by Zn anodes in aqueous electrolytes, paving the way toward practical and high‐performing ZIB systems.

## 3 Conclusion

This study demonstrates a novel and impactful approach to address the challenges faced by Zn anodes in aqueous ZIBs by constructing a tailored, high‐performance solid‐electrolyte interphase (SEI) layer using a cost‐effective ZnSiF_6_ additive. Through in‐depth electrochemical and microscopic characterization techniques combined with theoretical calculations, we elucidated the mechanism of action of this additive and established the remarkable stability of the engineered SEI. Our findings clearly demonstrate: **i) Solvation Structure Modification**: ZnSiF_6_ actively alters the solvation structure of Zn^2+^ ions in the electrolyte, facilitating rapid desolvation. **ii) Corrosion Inhibition**: The presence of ZnSiF_6_ leads to a significant reduction in corrosion of the Zn anode. The formation of a stable SEI effectively suppresses harmful side reactions, evidenced by significantly reduced byproduct formation (such as insulating ZHS) and a homogeneous, corrosion‐resistant morphology compared to the standard electrolyte system. **iii) Enhanced Kinetics: ZnSiF_6_** effectively reduces the activation energy for Zn^2+^ deposition/dissolution, accelerating charge transfer kinetics. **iv) Tailored Morphology**: ZnSiF_6_ modifies the nucleation and growth mechanism, shifting the deposition mode from 2D to a more uniform and dendrite‐suppressing 3D mode, resulting in a remarkably smooth, dense morphology of the deposited zinc. Stable. **v) Hierarchical SEI**: We characterized the ZnSiF_6_‐induced SEI in detail, confirming its unique structure: a porous framework of alkali zinc sulfate (ZHS) infused with conductive ZnF_2_. This hierarchical design, supported by XPS, STEM, and ToF‐SIMS, enables a remarkable synergy of fast Zn ion transport, corrosion resistance, and mechanical stability.

Our combined theoretical and experimental approach provided a comprehensive mechanistic understanding of how this unique SEI enables superior electrochemical performance in Zn||Zn symmetric cells, with extended cycling life exceeding 3000 h. Furthermore, full cell tests using a Zn||VO_2_ configuration, including a pouch cell demonstration, corroborated these benefits, exhibiting remarkable enhancements in both rate capability and long‐term cycling stability. This research underscores the effectiveness and immense potential of a rational design approach for developing stable, tailored SEIs for Zn anodes, effectively paving the way toward practical applications of aqueous ZIBs as safe, reliable, and high‐performance energy storage technologies.

## Conflict of Interest

There are no conflicts of interest to declare.

## Supporting information



Supporting Information

## Data Availability

The data that support the findings of this study are available in the supplementary material of this article.
