# Supplementary material for: Ultra‐Stable Aqueous Zinc Anodes: Enabling High‐Performance Zinc‐Ion Batteries via a ZnSiF6‐Derived Protective Interphase
Source: Adv Sci (Weinh). 2024 Oct 7;11(44):2407201. doi: 10.1002/advs.202407201 (PMC11600264; doi:10.1002/advs.202407201)
Supplement: Supplementary file 1 — Supporting Information [file ADVS-11-2407201-s001.docx]

**Ultra-Stable Aqueous Zinc Anodes: Enabling High-Performance Zinc-Ion Batteries via a ZnSiF_6_-Derived Protective Interphase**

Yongfeng Huang ^a,b^, Rongsheng Guo ^a^, Zejian Li ^a^, Jiajia Zhang ^a^, Wenbao Liu ^c,*^, Feiyu Kang ^a,b,*^

^a^ Institute of Materials Research, Tsinghua Shenzhen International Graduate School, Tsinghua University, Shenzhen 518055, China

^b^ School of Materials Science and Engineering, Tsinghua University, Beijing 100084, China

^c^ School of Environmental and Materials Engineering, Yantai University, Yantai 264005, China

*Corresponding authors and email addresses：

*wbliu@ytu.edu.cn* (W. Liu), [*fykang@mail.tsinghua.edu.cn* (Feiyu](mailto:fykang@mail.tsinghua.edu.cn%20(Feiyu) Kang).

**Experimental Procedures**

**VO_2_ Preparation Method:**

VO_2_(B) powder was prepared using the solvothermal method. First, 0.3 g of V_2_O_5_ powder was dissolved in 20 mL of deionized water, and 4 mL of 30% hydrogen peroxide was added under magnetic stirring. The mixture was stirred for 20 minutes until the solution turned orange-red. Then, 10 mL of this solution was added to a mixture of 50 mL of n-butanol and 30 mg of sodium dodecyl benzenesulfonate. The combined solution was poured into a 100 mL polytetrafluoroethylene-lined hydrothermal reactor and heated at 180 °C for 24 hours. After the reaction, the product was vacuum filtered, washed with deionized water and ethanol, and then dried at 60 °C for 12 hours to obtain black VO_2_(B) powder.

**Materials Characterization**:

High-resolution transmission electron microscopy (TEM) images were obtained using a FEI Tecnai G2 F30 microscope, operated at 300 kV. The aberration-corrected high-angle annular dark-field scanning TEM (HAADF-STEM) and integrated differential phase contrast STEM (iDPC-STEM) imaging were conducted on a double Cs-corrected cold-field-emission TEM (Thermo Fisher Spectra 300), operated at 300 kV with a convergence angle of 21.7 mrad. 3D Confocal Laser Microscope images were acquired using a Keyence VK-X200 (Japan). The Focused Ion Beam (FIB), Scanning Electron Microscopy (SEM), and Energy Dispersive X-ray Spectroscopy (EDX) capabilities were employed using the FEI Scios 2 DualBeam system. X-ray diffraction (XRD, Rigaku D/max-2500) employing Cu Kα radiation (λ=1.5418 Å) was utilized to examine the crystal structure over an angular range of 5°-90° (2θ). The surface/cross-section morphology and microstructure of modified electrolyte pellets were observed using scanning electron microscopy (SEM, HITACHI S4800, Japan), with elemental mapping patterns assessed by energy dispersive spectroscopy (EDS). X-ray photoelectron spectroscopy (XPS) measurements were conducted on a PHI 5000 Versa probe II spectrometer using a monochromatic Al Kα X-ray source. The 3D surface morphologies and surface roughness were analyzed through atomic force microscopy (AFM, Bruker Dimension Icon) tests. Time-of-flight secondary ion mass spectrometry (TOF-SIMS) was performed using a Nano TOF-2 instrument (ULVAC-PHI, Japan) equipped with a 30 kV Bi^3+^ beam cluster primary-ion gun and an Ar+ beam (3 keV, 100 nA) for depth profiling at a sputtering rate of 0.1 nm s-1. Raman spectral analysis was conducted at room temperature using a Horiba LabRAM HR800 miniature laser confocal Raman spectrometer (France), employing a 532 nm laser. In situ optical observations were conducted to visualize Zn plating/stripping in various electrolytes using a polytetrafluoroethylene cell with a transparent quartz window (YM710TR).

**Electrochemical Characterization**:

Zn symmetric cells were constructed by placing glass fiber separators between commercial Zn foil (80 µm) in CR2032-type cells filled with either ZSO or ZSO-ZnSiF_6_ electrolyte. Electrochemical analyses, including linear sweep voltammetry (LSV), cyclic voltammetry (CV), and galvanostatic charge/discharge (GCD) tests, were carried out using a multichannel electrochemical station VMP3 (BioLogic Science Instruments, France), covering a frequency range from 10 kHz to 0.01 Hz, with an alternating current (AC) amplitude of 10 mV. The LSV procedure was implemented in a two-electrode configuration, employing Ti foil as the working electrode and Zn foil as the reference and counter electrodes. This configuration was scanned from the open circuit potential to the designated polarization limits in both positive and negative directions, maintaining a consistent scan rate of 1 mV s^-1^. CV and GCD measurements were accurately recorded following an initial two-cycle conditioning phase. Further, linear polarization studies were executed utilizing an advanced three-electrode system. This setup comprised a Zn foil as the working electrode, a Ti plate as the counter electrode, and a Hg/HgSO_4_ electrode as the reference.

To assess the electrochemical impedance spectrum (EIS) of Zn||Zn symmetric cells, we employed a VMP3 electrochemical workstation (Bio-Logic company, France). These measurements were taken over a frequency range of 0.01 Hz to 100 kHz with a 10 mV oscillation voltage. Additionally, chronoamperometry (CA) tests were executed with the three-electrode configuration, applying an overpotential of -150 mV throughout 300 s. The cycling performance of the cells was evaluated using a LAND testing system (CT2001A, Wuhan Land Electronic Co. Ltd., China). For these experiments, Zn foils, each with a surface area of 1.13 cm2 and a thickness of 80 or 20 µm, were utilized in both Zn||Zn and Zn||Cu cell configurations. These rate and long-term cycling tests for full batteries were conducted post-activation cycles. All electrical tests were performed at 25°C.

**COMSO**L

The finite element simulation was performed using the Tertiary Current Distribution module and Phase Field module in COMSOL 5.6. This coupled approach simulated the distribution of electrolyte ion concentration, current, and morphological changes of the coating during a complete charge-discharge cycle in aqueous zinc-ion batteries. Within a simulation area of 1.5 µm x 2 µm, a dynamic mesh was utilized to ensure the accuracy of transient simulation data. During constant current charging, localized reduction and deposition of Zn ions occurred at the electrode region alongside the competing hydrogen evolution reaction (HER). During continuous current discharging, the electrochemical corrosion and dissolution of Zn metal occurred. The hydrogen gas production in a cycle was determined by integrating the HER current component during the charging process.

The simulation was grounded in fundamental physical relationships of electrochemical reactions: the electrode material's surface equilibrium potential followed the Nernst equation, reaction kinetics were based on the Butler-Volmer equation, dilute substance diffusion in the electrolyte obeyed Fick's laws, and charged substance diffusion followed the Nernst-Einstein relation. Through coupling the electrochemical current distribution, mass transport processes, and electrode morphological changes, the simulation yielded the electrolyte potential distribution, current distribution, and morphological variations of the coating during charge-discharge cycles. The solver was based on free triangular meshing, employing the PARADISO (parallel sparse direct solver) and Newton's method for convergence with a relative tolerance set to 1E^-6^ to ensure result accuracy.

The electrode surface reactions adhere to the Butler-Volmer (BV) equation:

$$i_{\mathrm{loc}}=i_{0}\left( \exp\left( \frac{\alpha_{a}F\eta}{RT} \right)-exp\left( \frac{-\alpha_{c}F\eta}{RT} \right) \right)$$

The equilibrium potential follows the Nernst equation:

$$E_{\mathrm{cq}}=-\frac{\Delta G}{nF}$$

$$E_{\mathrm{cq}}=E_{cq,ref}-\frac{RT}{nF}\ln\prod_{i} \left( \frac{a_{i}}{a_{i,ref}} \right)^{\nu_{i}}$$

The transfer of dilute substances adheres to Fick's laws:

$$\mathbf{N}_{i}=\mathbf{J}_{i}=-D_{i}\nabla c_{i} \frac{\partial c_{i}}{\partial t}+\nabla\cdot\mathbf{N}_{i}=R_{i,tot}$$

The electro-migration transport follows the Nernst-Einstein relation:

$$\mathbf{N}_{i}=-D_{i}\nabla c_{i}-z_{i}u_{m,i}Fc_{i}\nabla\phi_{l}+\mathbf{u}c_{i}=\mathbf{J}_{i}+\mathbf{u}c_{i}$$

**DOD Calculation[1]**

The depth of discharge (DOD) was calculated considering both the thickness of the zinc foil and the deposited zinc mass. For a zinc foil of thickness t (µm), the total theoretical capacity per unit area Q_total_ (mAh/cm^2^) is calculated as:

$$Q_{\mathrm{total}}=t\times\rho\times E$$

Where:

$t$ is the thickness of the zinc foil (µm)

$\rho$ is the density of zinc (7.14 g/cm^3^)

$\text{E}$ is the theoretical specific capacity of zinc (820 mAh/g)

The DOD is then calculated using the formula:

$$\mathrm{DOD}=(\frac{Q_{\mathrm{discharge}}}{Q_{\mathrm{total}}})\times100\%$$

Where $Q_{discharge}$ is the discharged capacity per unit area of the battery (mAh/cm^2^).

Then

$$\mathrm{DOD}=Q_{\mathrm{discharge}}/(0.58548\times t_{\mu m})$$

For example, in our DOD experiments, we utilized a 20 µm thick zinc foil and achieved a discharge capacity of 10 mAh/cm^2^. Using these values, our revised calculation yields a DOD of 85.4%.

**MD and DFT**

Molecular dynamics (MD) simulations were applied to investigate the solvation structures for ZnSO_4_/ZnSiF_6_ aqueous solutions. The considered electrolyte systems consisting of 200 ZnSO_4_, 10 ZnSiF_6_, and 5500 H_2_O molecules were constructed into one cubic simulation box. All MD simulations were carried out by the Forcite module with the COMPASS III force field[2] in MS 2020. Van der Waals and Coulomb interactions were respectively considered by atom-based and Ewald methods with a cut-off value of 12.5 Å. Equations of motion were integrated with a time step of 1 fs. After energy minimization, the electrolyte system was fully relaxed under periodic boundary conditions for 400 ps in the NPT (P = 1 atmosphere, T = 298.0 K) ensemble using the Nose thermostat and Berendsen barostat, which was long enough for system temperature, potential, and total energy to stabilize. After reaching the equilibrium state, another 400 ps simulation under the NVT ensemble was performed to extract trajectory and data for radial distribution function (RDF) and coordination number (CN) calculations. The dynamic trajectory for each system was outputted at an interval of 4 ps.

The coordination number Ni of atom i in the first solvation shell surrounding Zn^2+^ was calculated as:

*N_i_* = 4π*ρ*$\int_{0}^{R_{M}} g\left( r \right)r^{2}dr$

where *R_M_* is the distance of the first minimum following the first peak in the RDF *g(r)*, and *ρ* is the number density of atom *i*[3].

The solid-liquid interface calculations for 2M ZnSO_4_ and ZnSO_4_/ZnSiF_6_ aqueous solution on Zn (0 0 2) surface were conducted with COMPASS Ⅲ force field using the Forcite tools in MS 2020.

The dimensional lengths of the rectangular Zn (002) slab are 42.64 Å × 41.54 Å in plane, which contains 5 layers of Zn. The 2 M ZnSO_4_ aqueous solution model is composed of 100 ZnSO_4_ and 2750 H_2_O molecules in a rectangular box with length scales of 42.64 Å × 41.54 Å × 45.65 Å. The ZnSO_4_/ZnSiF_6_ aqueous solution contains 100 ZnSO_4_, 5 ZnSiF_6_, and 2750 H_2_O molecules in one rectangular box with length scales of 42.64 Å × 41.54 Å × 45.83 Å. After geometry optimization, the two aqueous solutions were placed on the Zn (002) surface, respectively. The electrode potential was controlled with the charge density of the top layer zinc metal surface by 0.1 e/Zn atom[4].

All MD calculations were performed under the NVT ensemble (T=298.0 K) with a time step of 1 fs and a total simulation time of 400 ps, during which simulation trajectories were recorded every 2000 steps. The running time was long enough for the system energy and temperature to reach a stable state. The temperature was controlled by a Nose-Hoover thermostat. The Ewald scheme and atom-based cutoff method (i.e., a radius of 12.5 Å) were applied to treat electrostatic and van der Waals (vdW) interactions, respectively. The concentration profiles of Zn^2+^, SO_4_^2-^, H_2_O, and SiF_6_^2-^ along the perpendicular direction were analyzed. The solid-liquid interfaces snapshots were displayed.

The adsorption calculations for H_2_O and SiF_6_^2-^ adsorption on top sites of Zn (002) and (101) surfaces were separately performed within the framework of density functional theory (DFT) as implemented in the Vienna Ab-initio Simulation Package (VASP) code using the projector augmented wave method with the Perdew-Burke-Ernzerhof (PBE) exchange-correlation functional[5]. The influence of vdW interactions is considered by using a modified version of vdW-DF, referred to as "optB86b-vdW" [6]. The projector augmented wave potentials were used with an energy cutoff of 600 eV. The lattice constraints for the Zn (002) slab, composed of 4 layers of Zn, are 10.66 Å × 10.66 Å. The dimensional length for the Zn (101) slab is 11.24 Å × 10.66 Å. There exists a vacuum layer larger than 20 Å perpendicular to the surface plane. A 4 × 4 × 1 Monkhorst method k-mesh was used for geometry optimization, during which the bottom layer of the Zn substrate was fixed. Energy convergence of 1.0 × 10^-4^ meV/atom was ensured during the self-consistent field calculations, and the convergence criteria for the atomic forces were 0.01 eV/Å. After structure optimization, one H_2_O and SiF_6_^2-^ were respectively adsorbed upon the top surface sites of Zn (002) and (101). Geometry optimization was then performed for each adsorption system, followed by a static calculation. The k-meshes were doubled for the single-point calculation.

The adsorption energies *E_ad_* were calculated according to the following equation:

$$E_{ad}=E_{total}-E_{solvent}-E_{slab}$$

Where *E_total_*, *E_solvent_*, and *E_slab_* are the energies of the adsorption system, adsorbed solvent (i.e., H_2_O or SiF_6_^2-^), and Zn (002) or (101) slab, respectively.


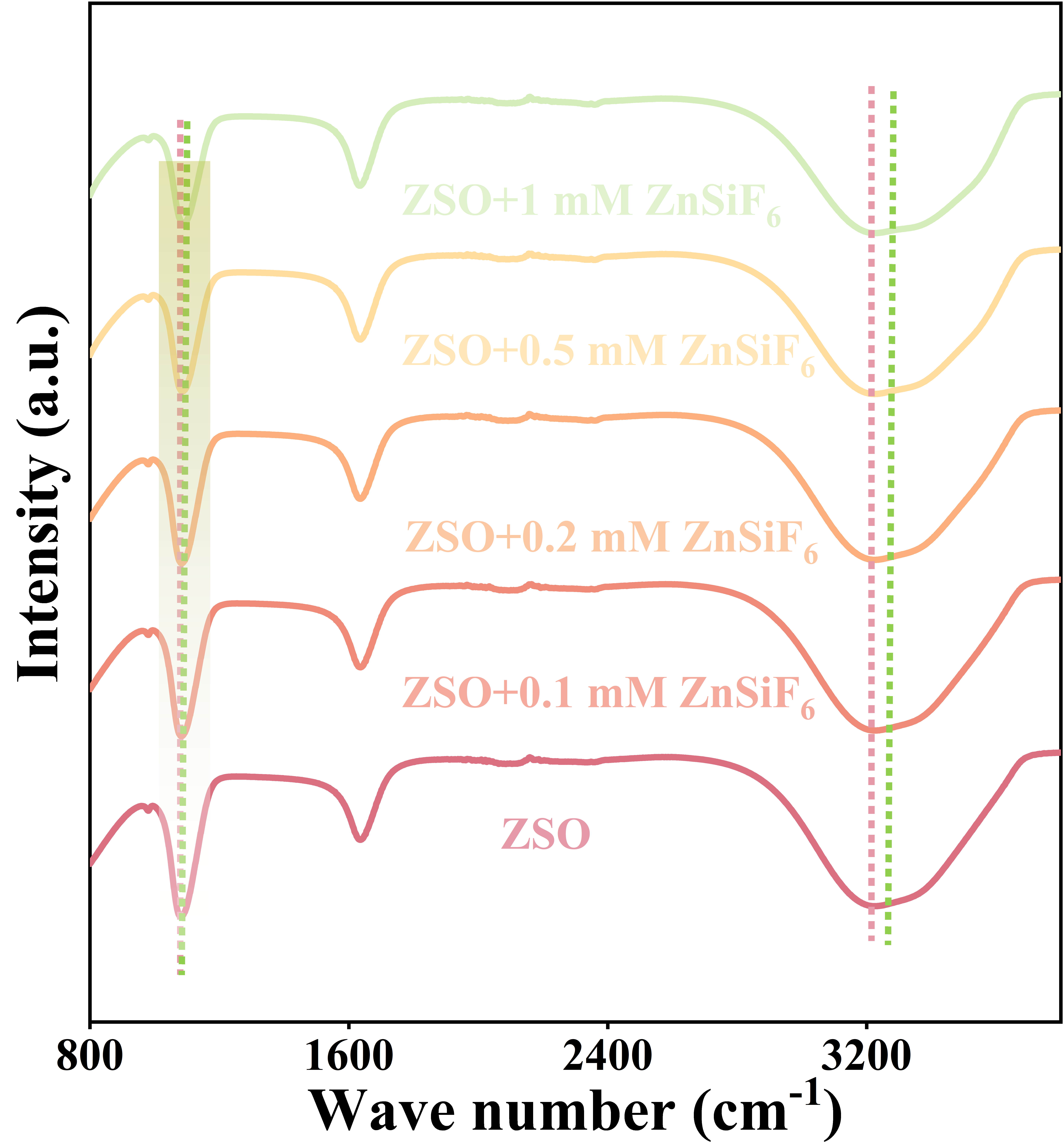


**Figure S1.** FT-IR spectra of the ZSO-ZnSiF_6_ electrolyte with varying concentrations.


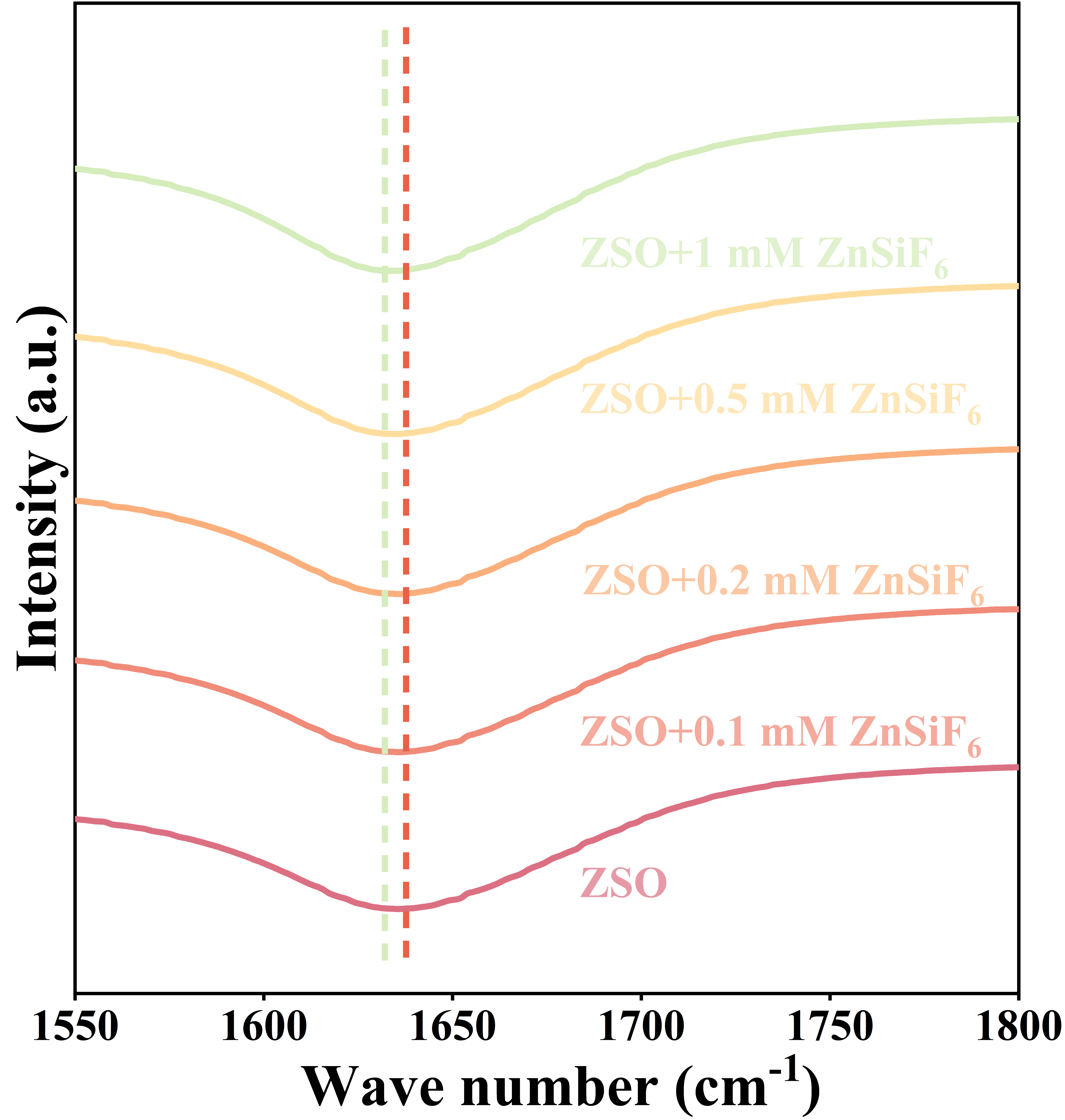


**Figure S2.** The bending vibration of OH^-^ in the FT-IR spectra of the ZSO-ZnSiF_6_ electrolyte with varying concentrations.


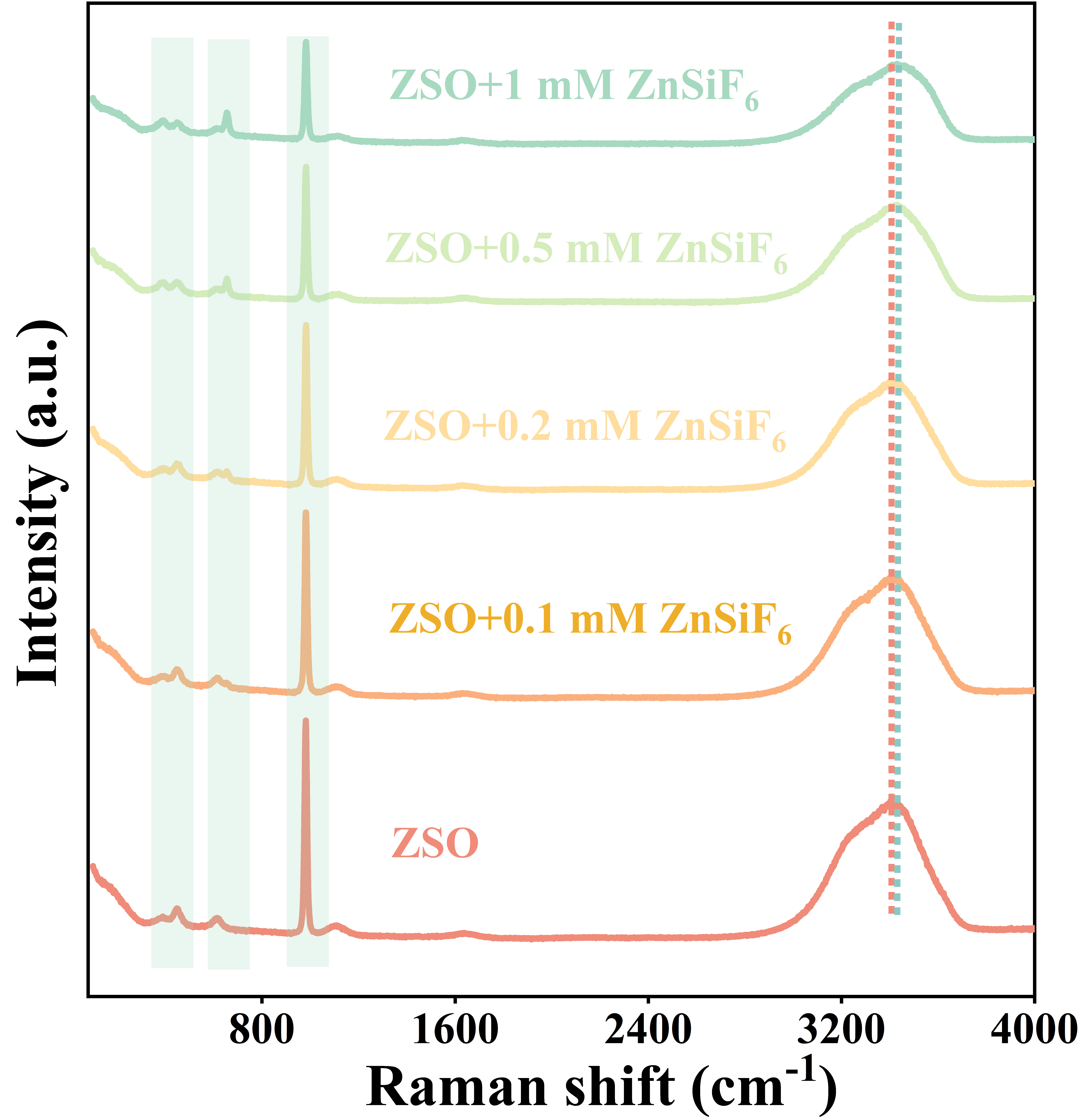


**Figure S3.** Raman spectra of the ZSO-ZnSiF_6_ electrolyte with varying concentrations.


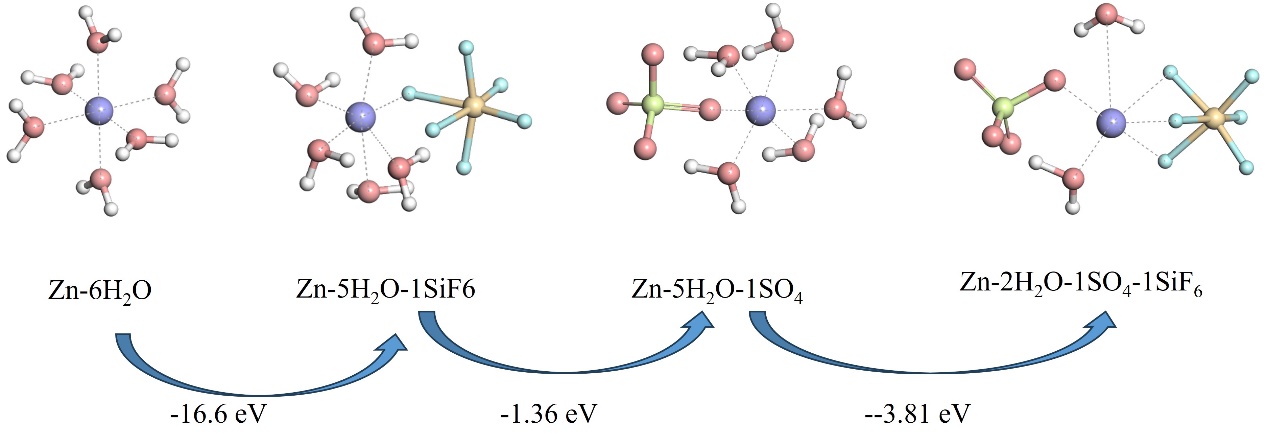


**Figure S4.** The schematic diagram for free energies in the ZSO-ZnSiF_6_ electrolyte.


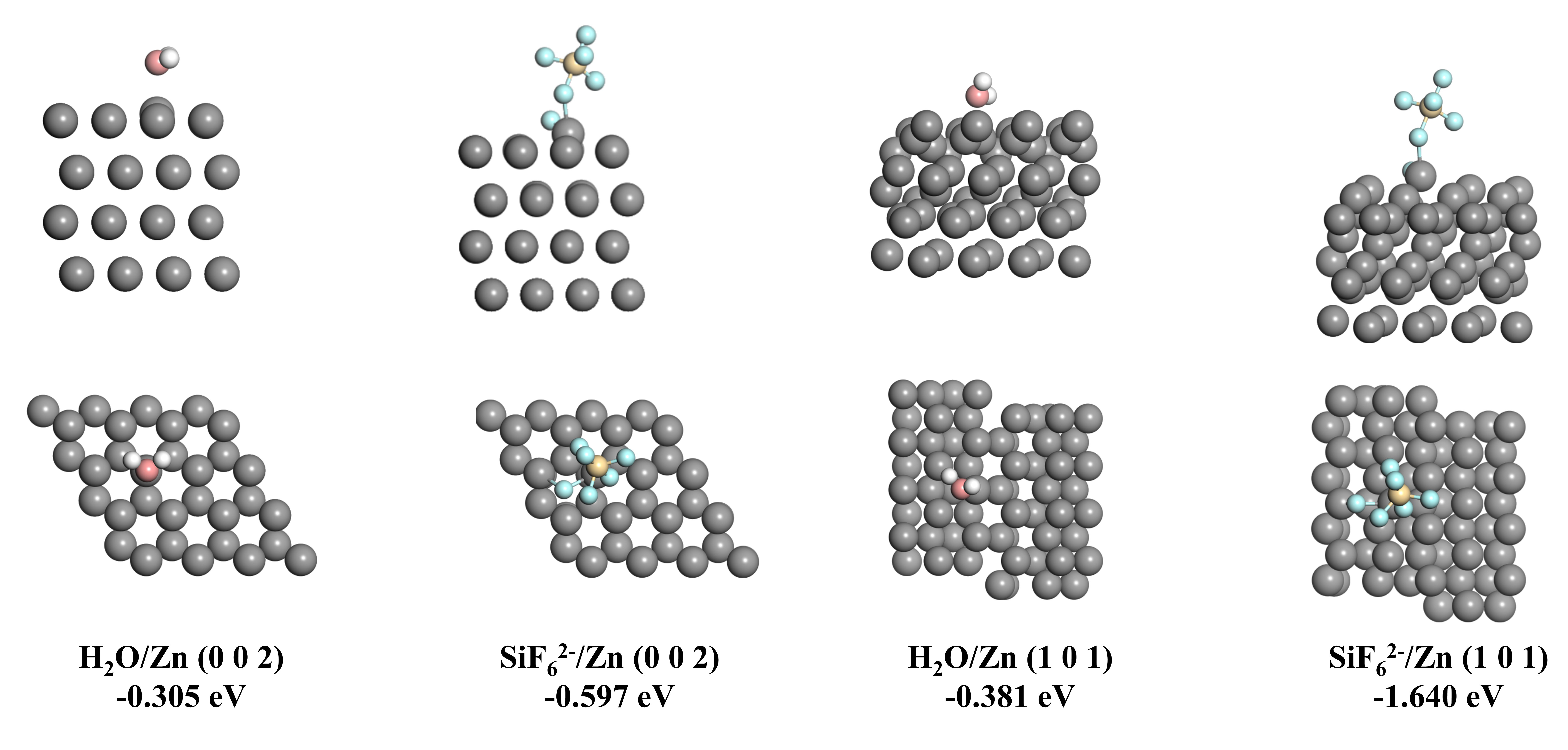


**Figure S5.** Adsorption energies of H_2_O and SiF_6_^2-^ molecules on Zn(002), (101) respectively.

**
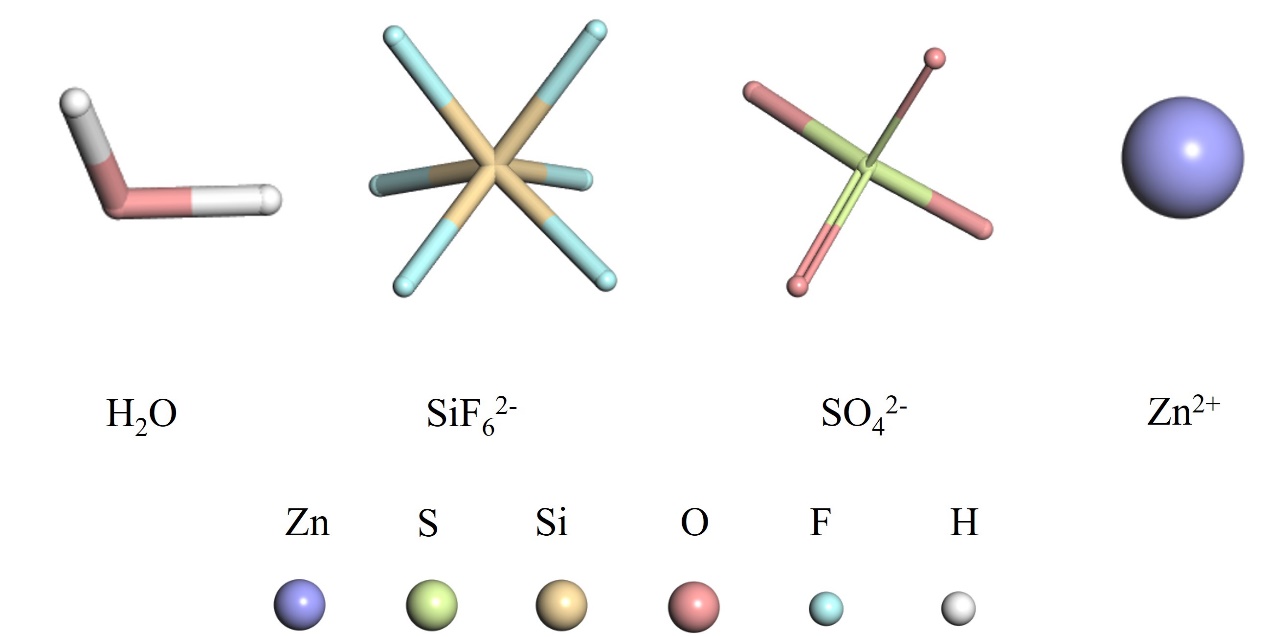
****Figure S6.** Representative snapshots of the ZSO-ZnSiF_6_ electrolyte. **
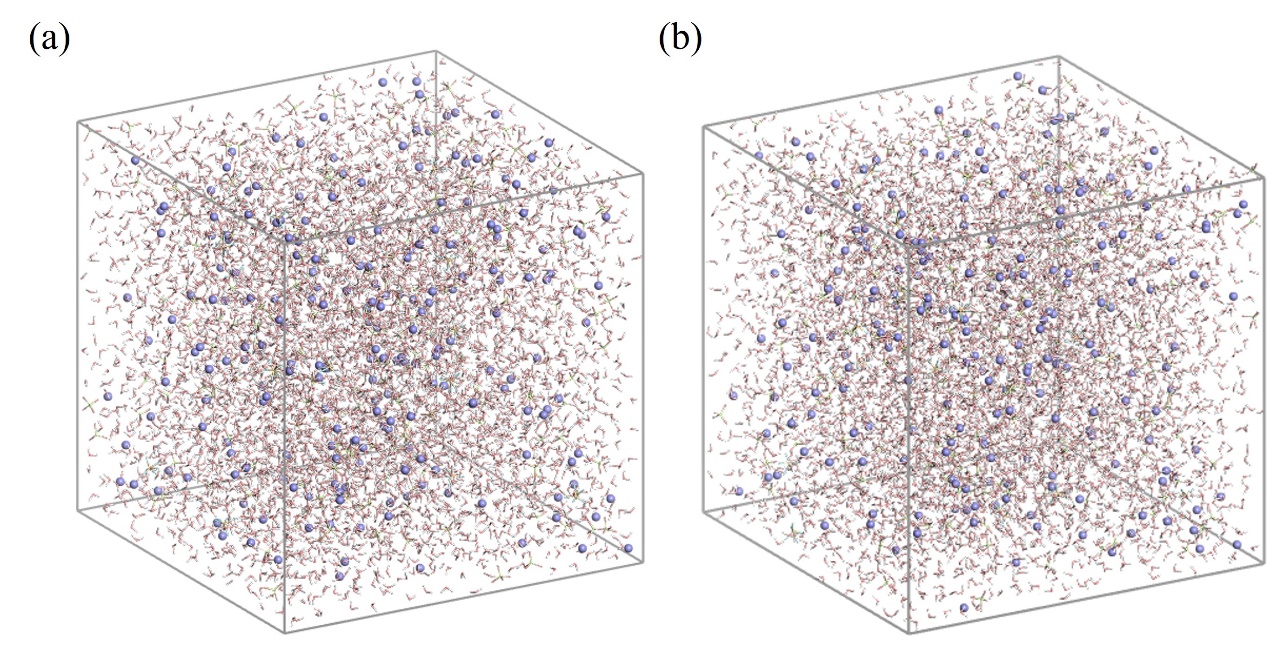
** **Figure S7.** MD of a) ZSO and b) ZSO-ZnSiF_6_ electrolyte.


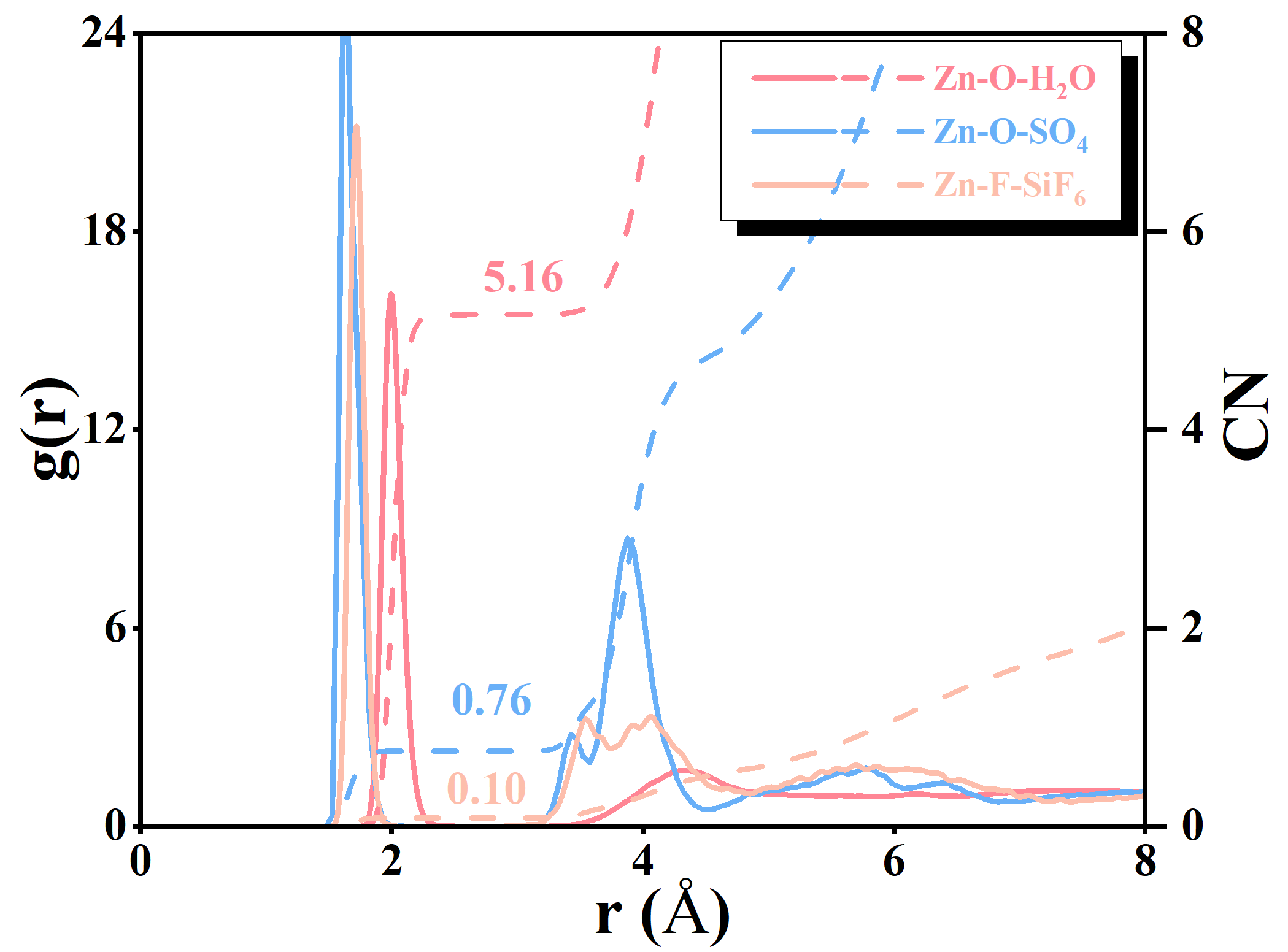


**Figure S8.** The radial distribution functions of the ZSO-ZnSiF_6_ electrolyte.


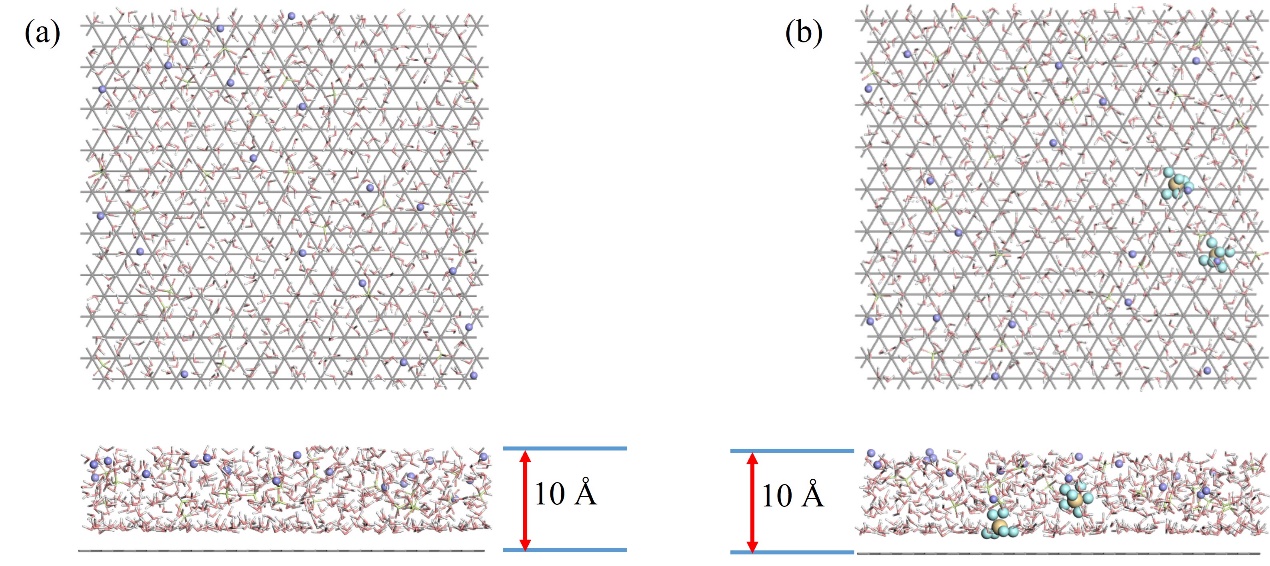


**Figure S9.** Top layer is charged by 0.1 e/atom (a) ZSO electrolytes on Zn(0 0 2) surface and (b) ZSO-ZnSiF_6_ electrolytes on Zn(0 0 2) surface.


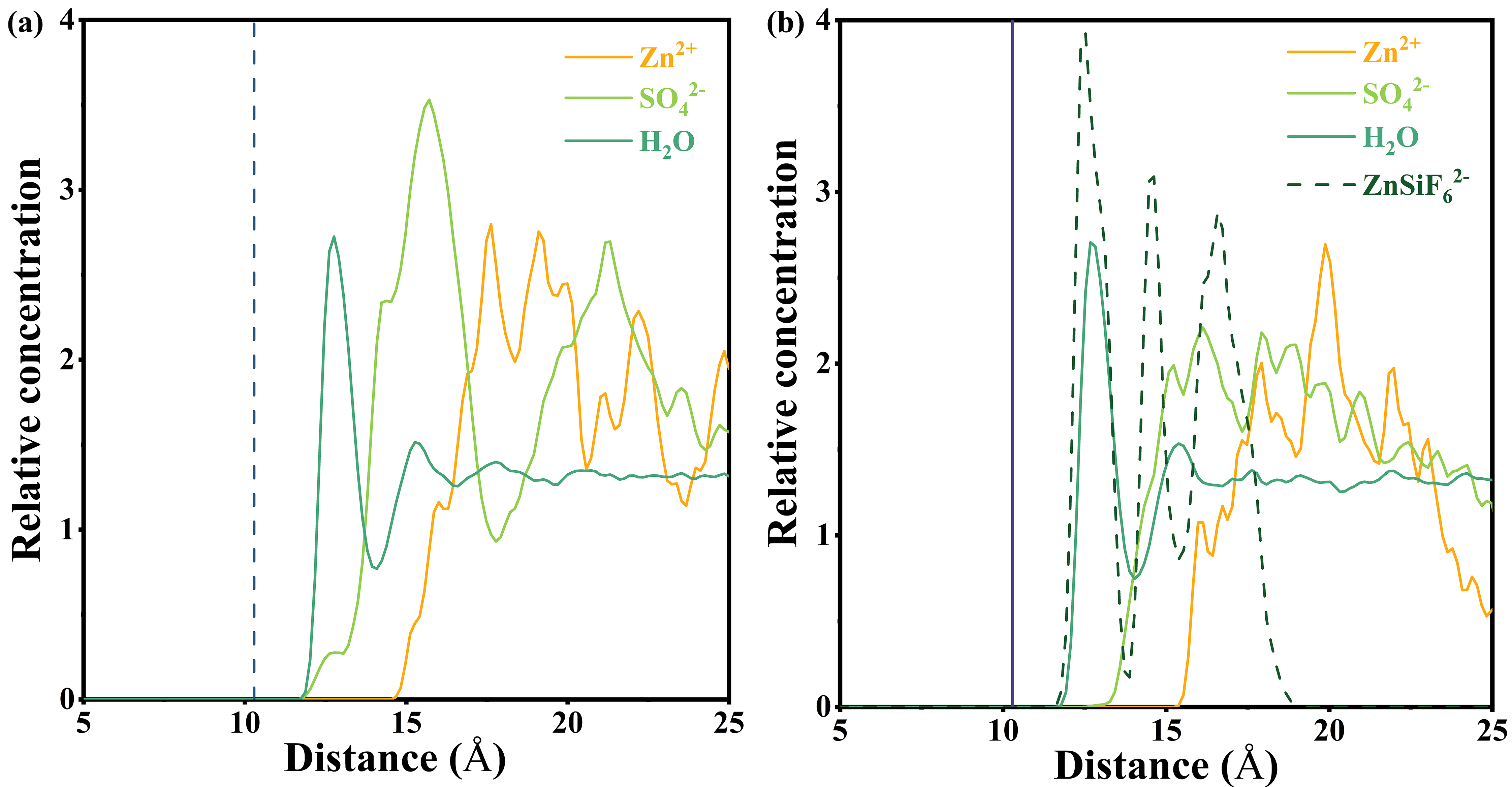


**Figure S10.** Electrolytes on Zn(0 0 2) surface relative concentration (a) ZSO and (b) ZSO-ZnSiF_6_.


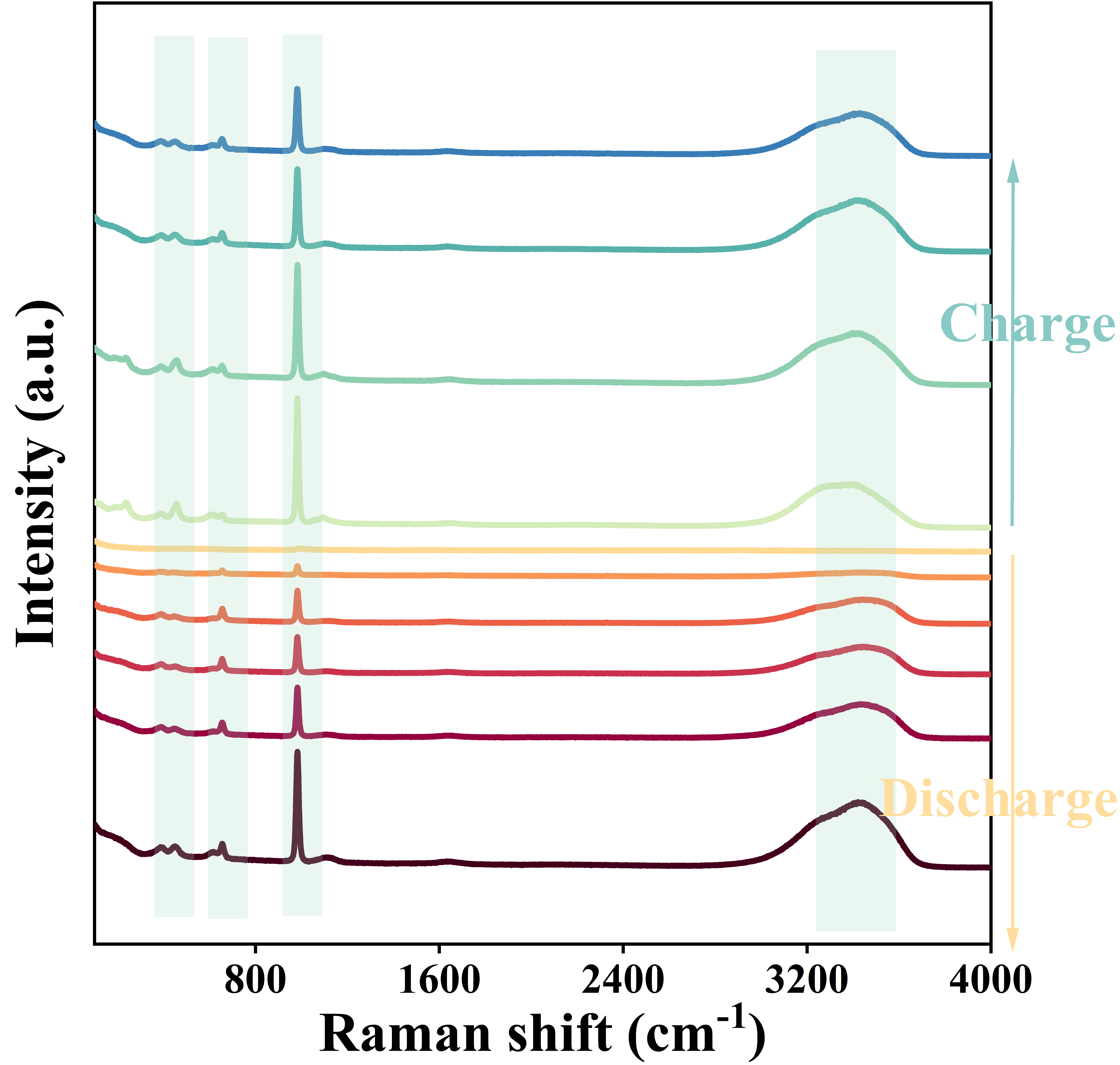


**Figure S11.** In-situ Raman spectra of the ZSO-ZnSiF_6_ electrolyte.


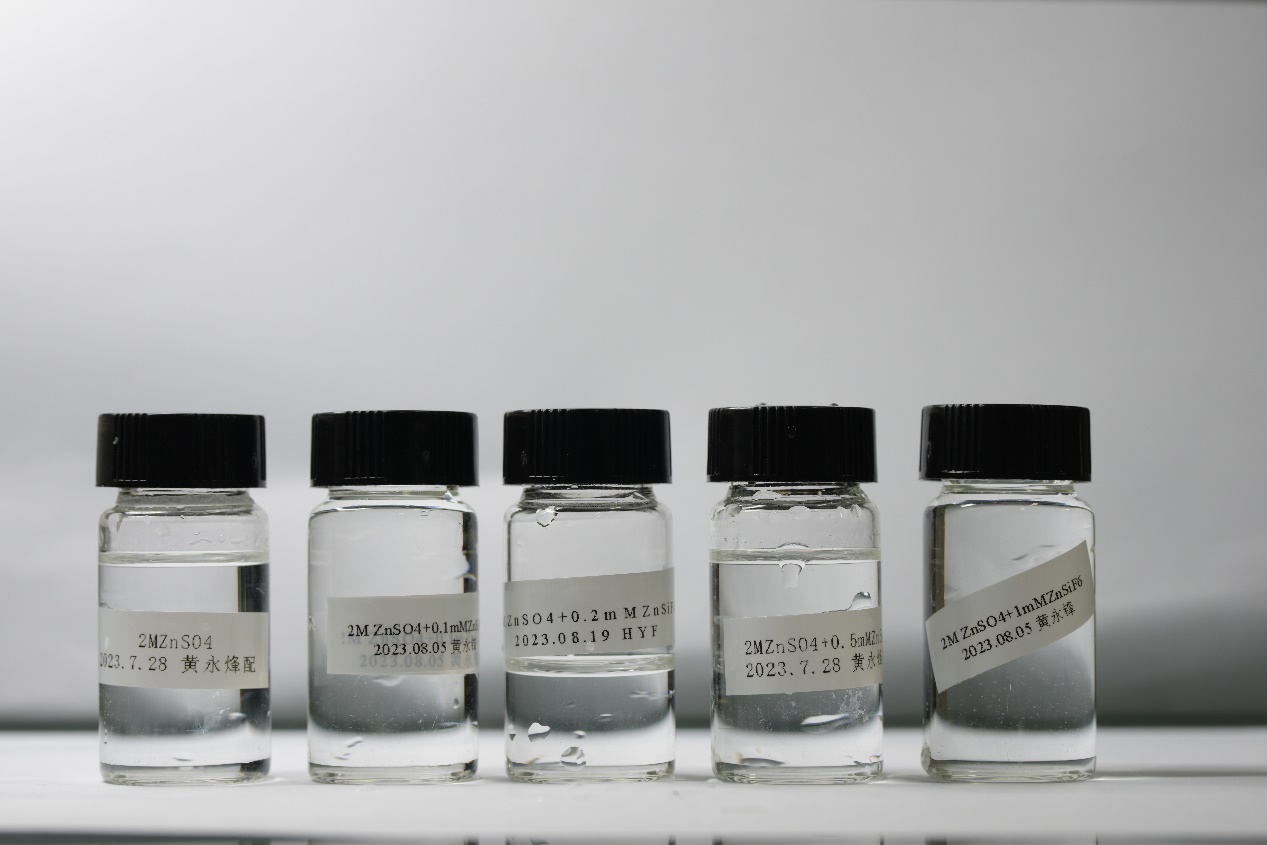


**Figure S12.** Digital photographs of the ZSO-ZnSiF_6_ electrolytes at different concentrations.


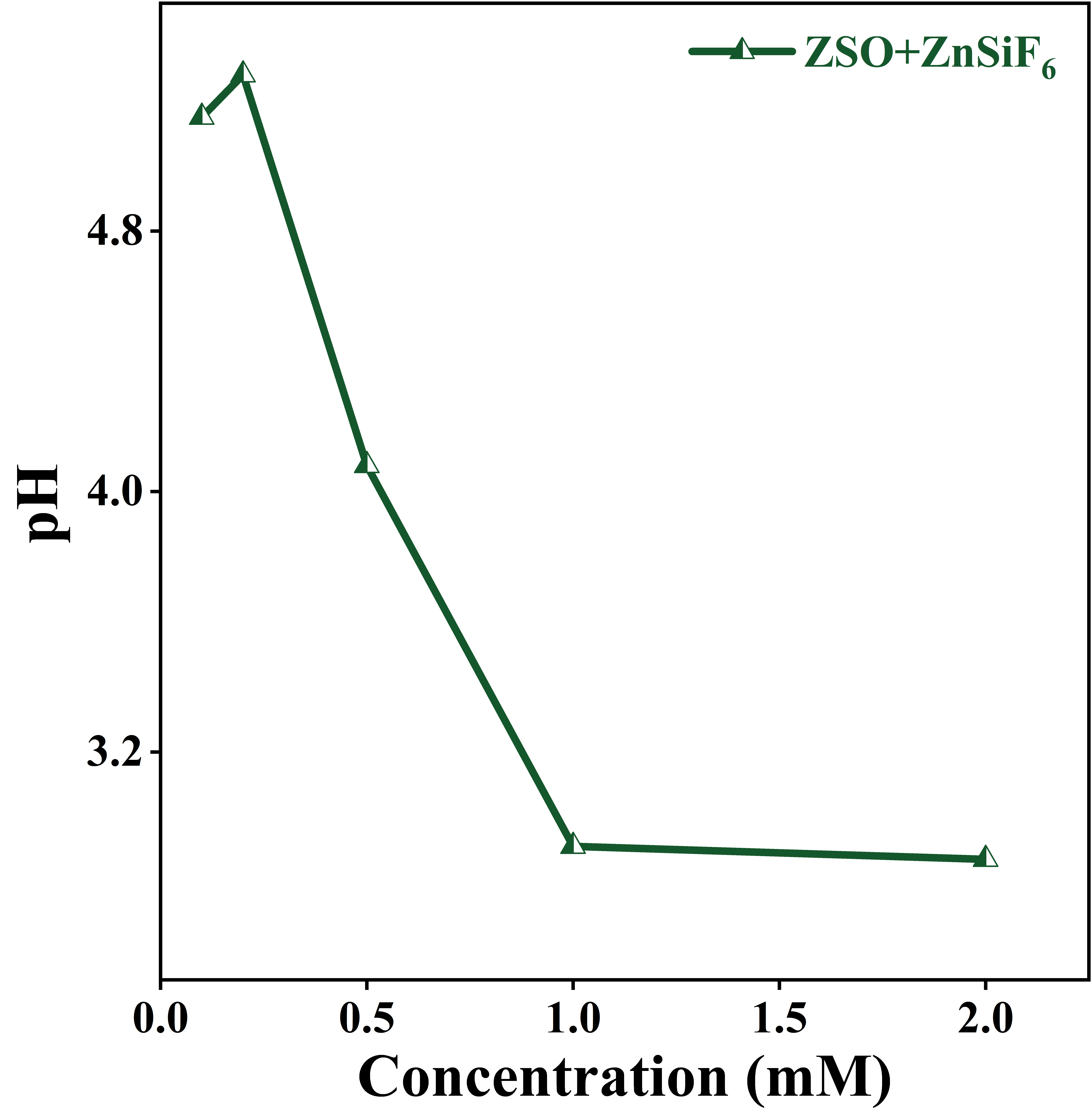


**Figure S13.** pH of the ZSO-ZnSiF_6_ electrolytes with different concentrations.


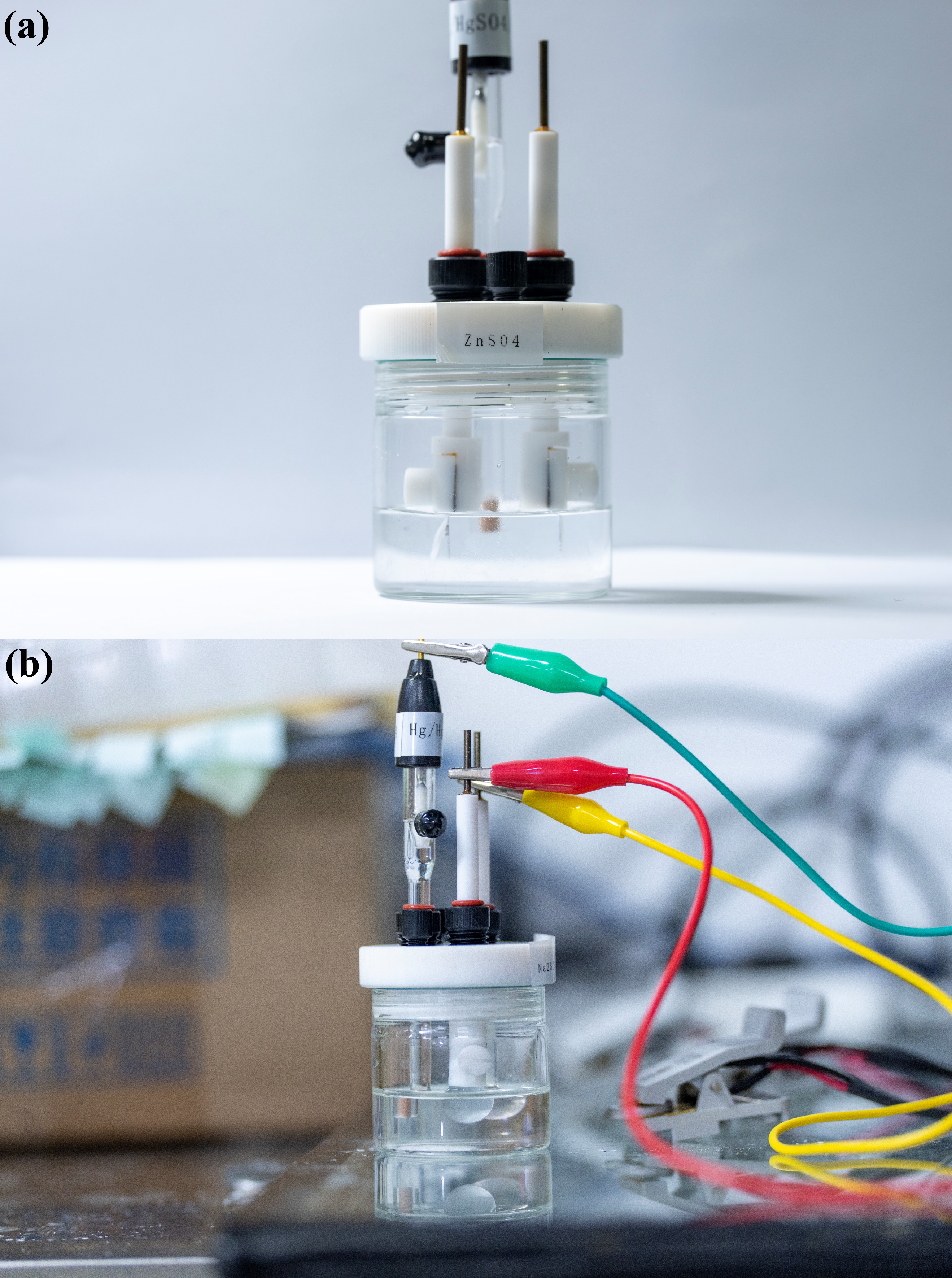


**Figure S14.** Digital photographs of trielectrode: a Zn foil as the working electrode, a Ti plate as the counter electrode, and a Hg/HgSO_4_ electrode as the reference.

**
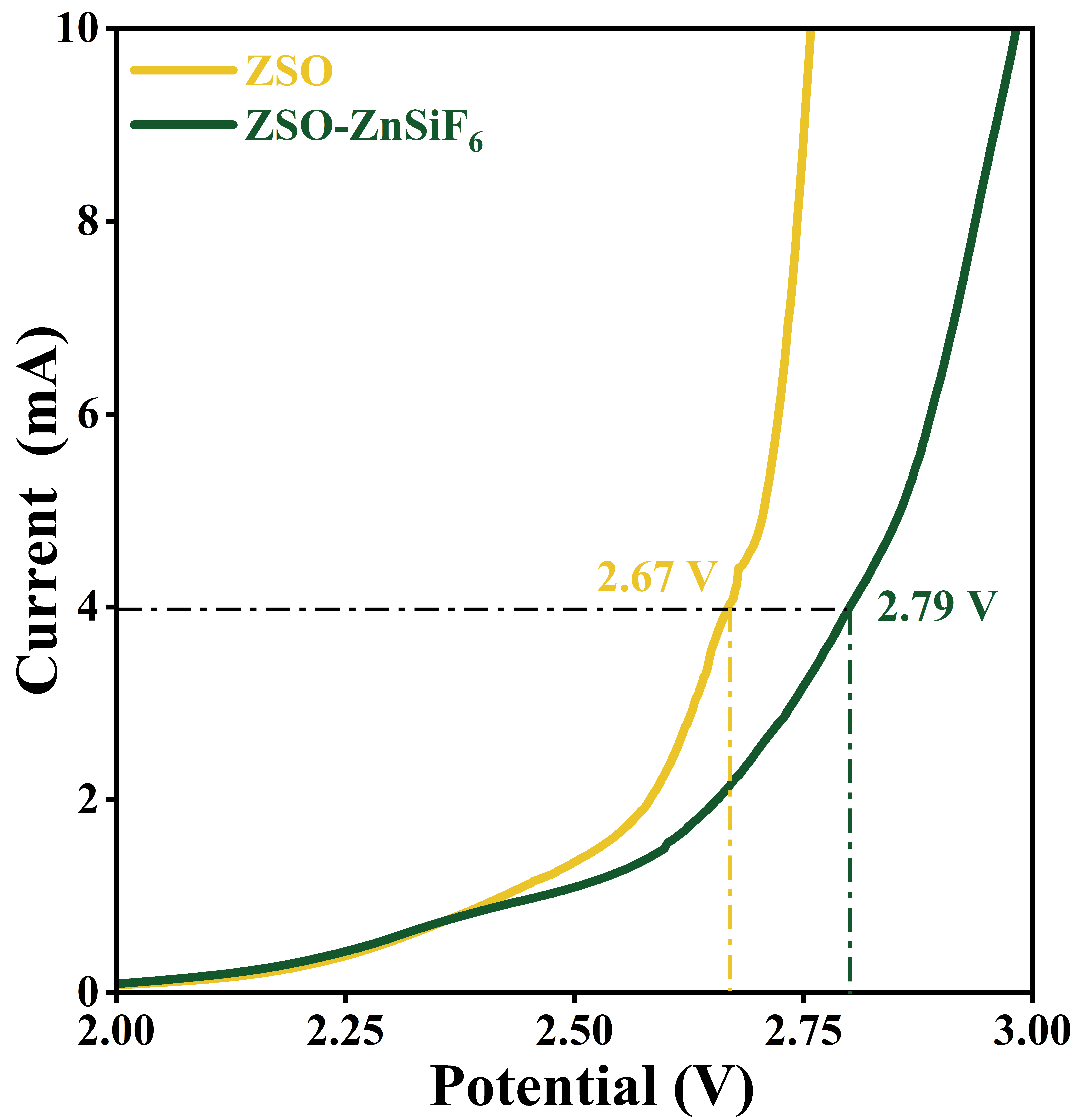
**

**Figure S15.** LSV curves of Zn||Ti batteries in the ZSO and ZSO-ZnSiF_6_ electrolyte.


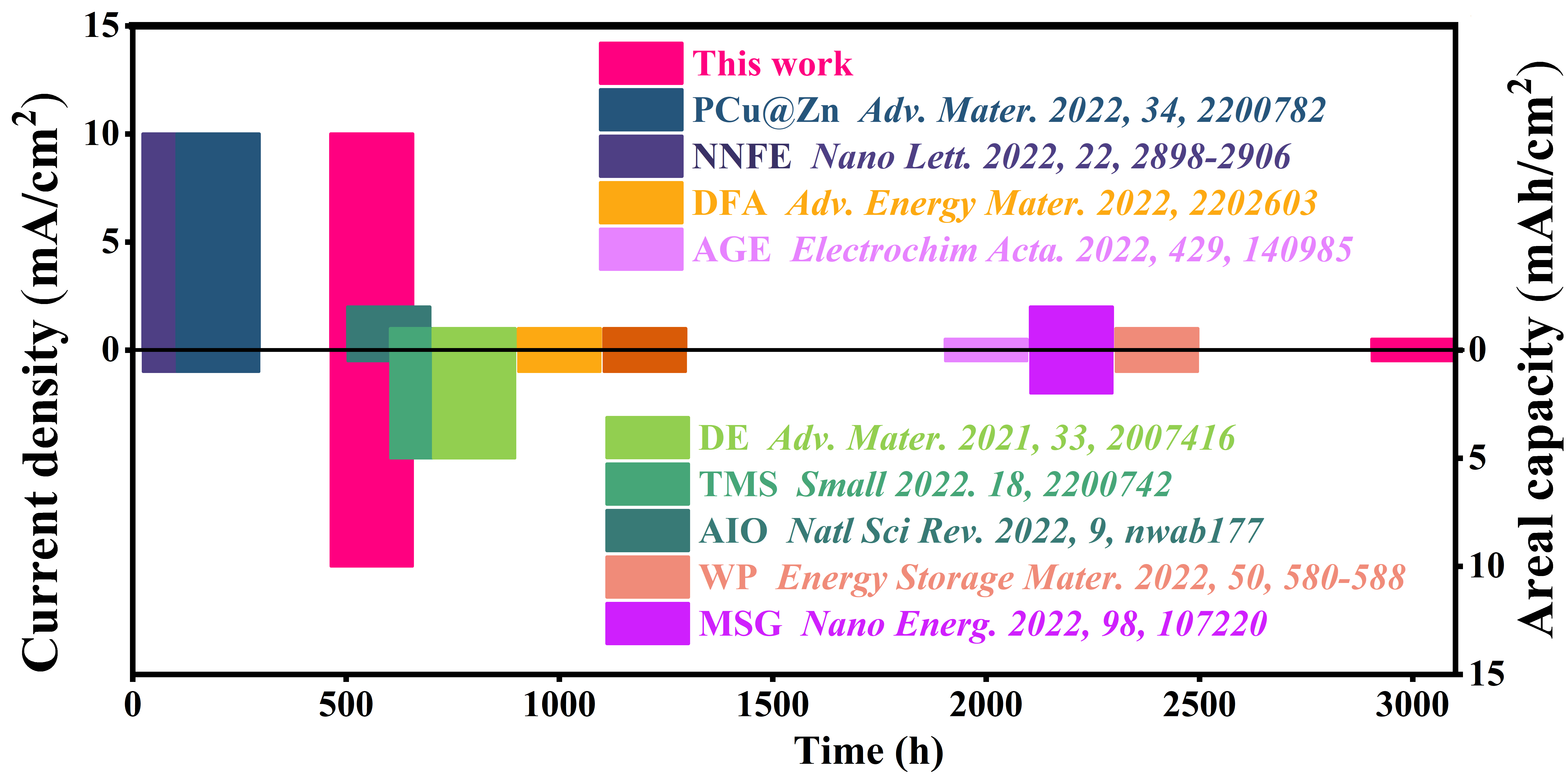


**Figure S16.** Comparison of the cycle life and areal capacity for Zn||Zn symmetric cells using various electrolyte additives[7].


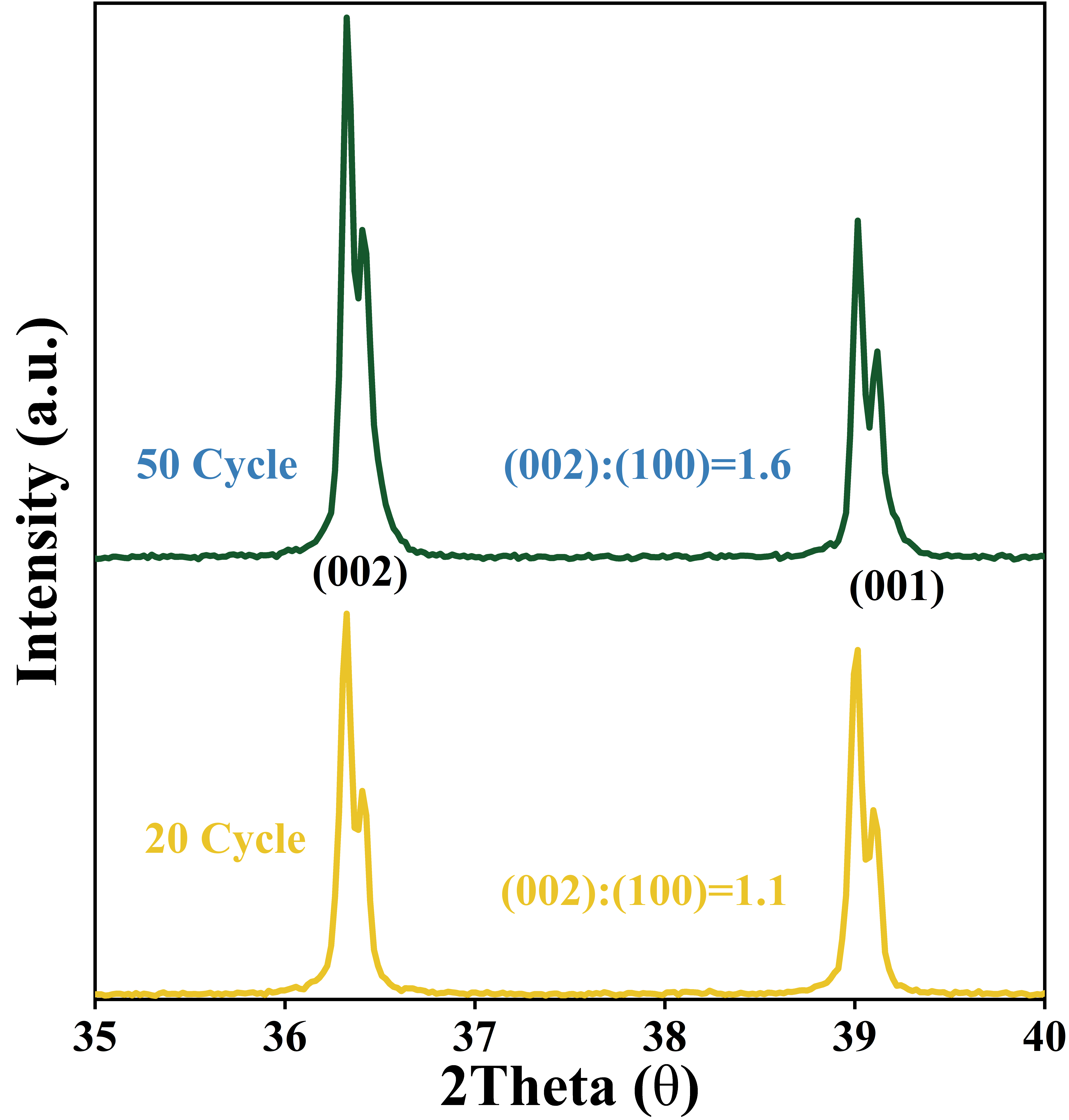


**Figure S17.** XRD of (002)/(101) after Zn foil cycled in the ZSO and ZSO-ZnSiF_6_ electrolyte for 20 and 50 times.
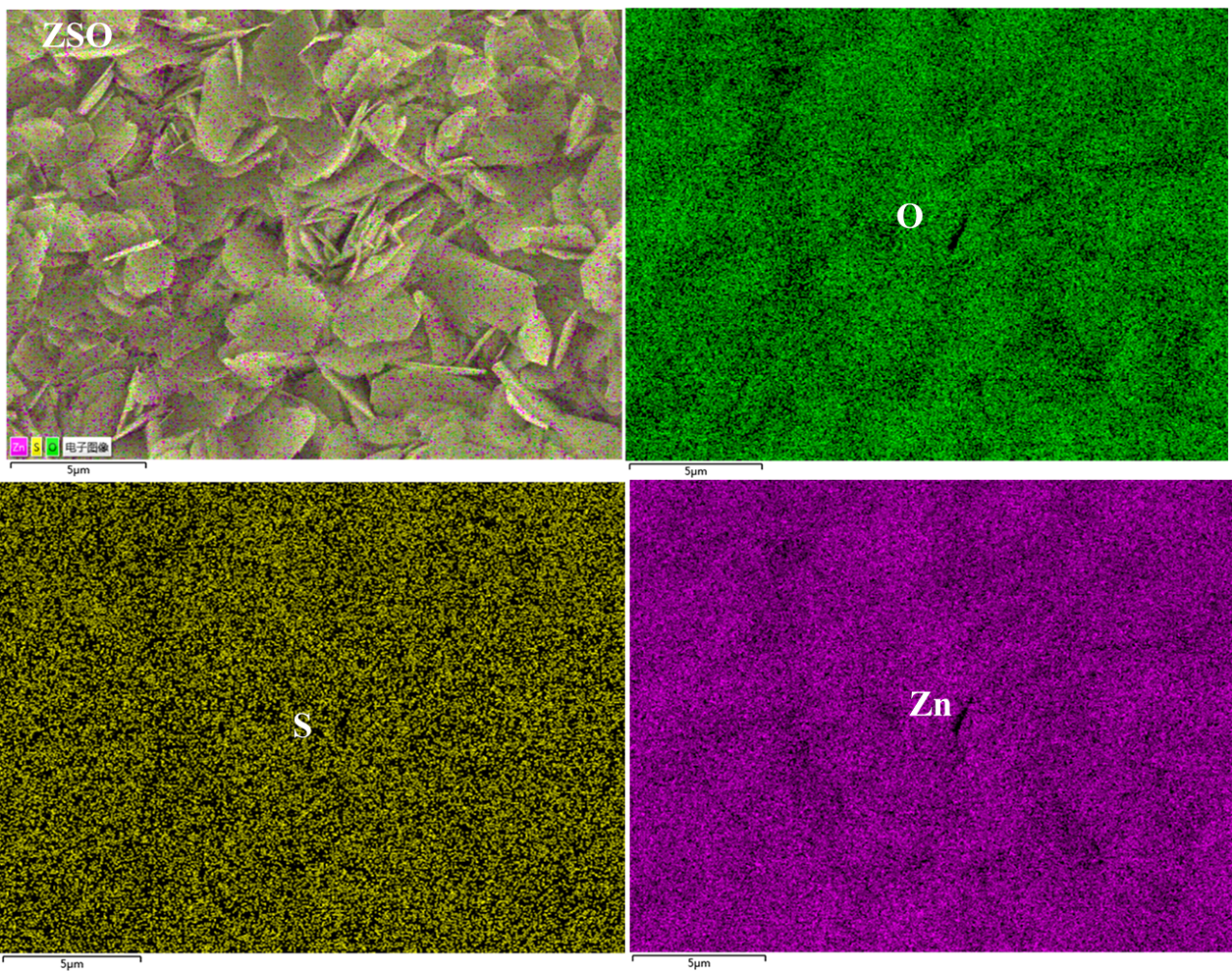


**Figure S18.** SEM-Mapping of Zn anode after 20 cycles in the ZSO electrolyte.
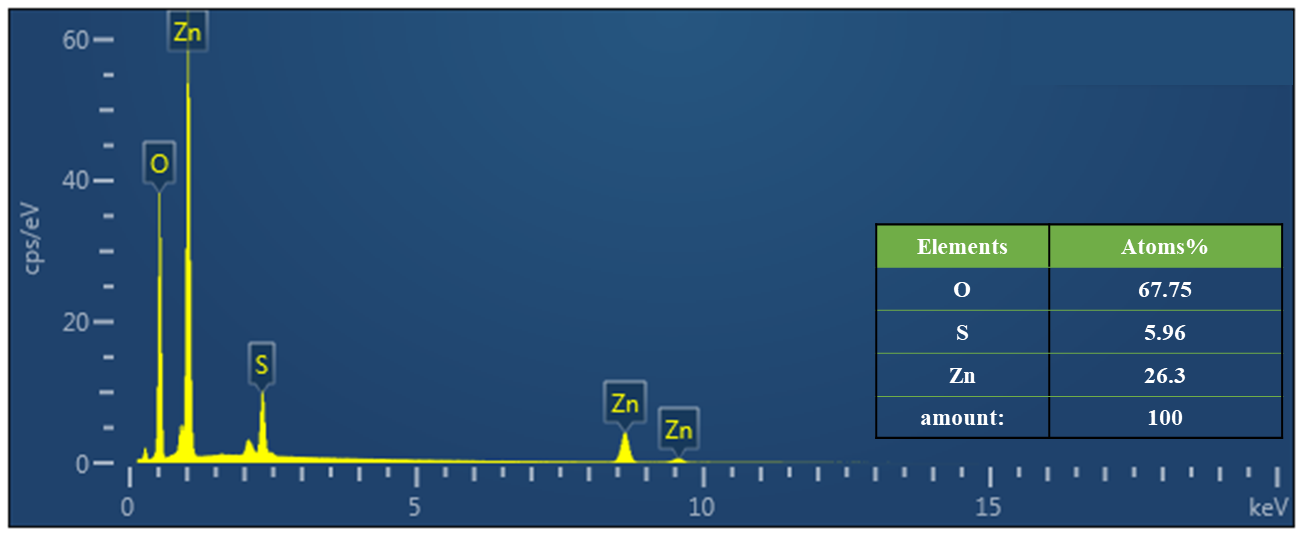


**Figure S19.** SEM-EDS of Zn anode after 20 cycles in the ZSO electrolyte.


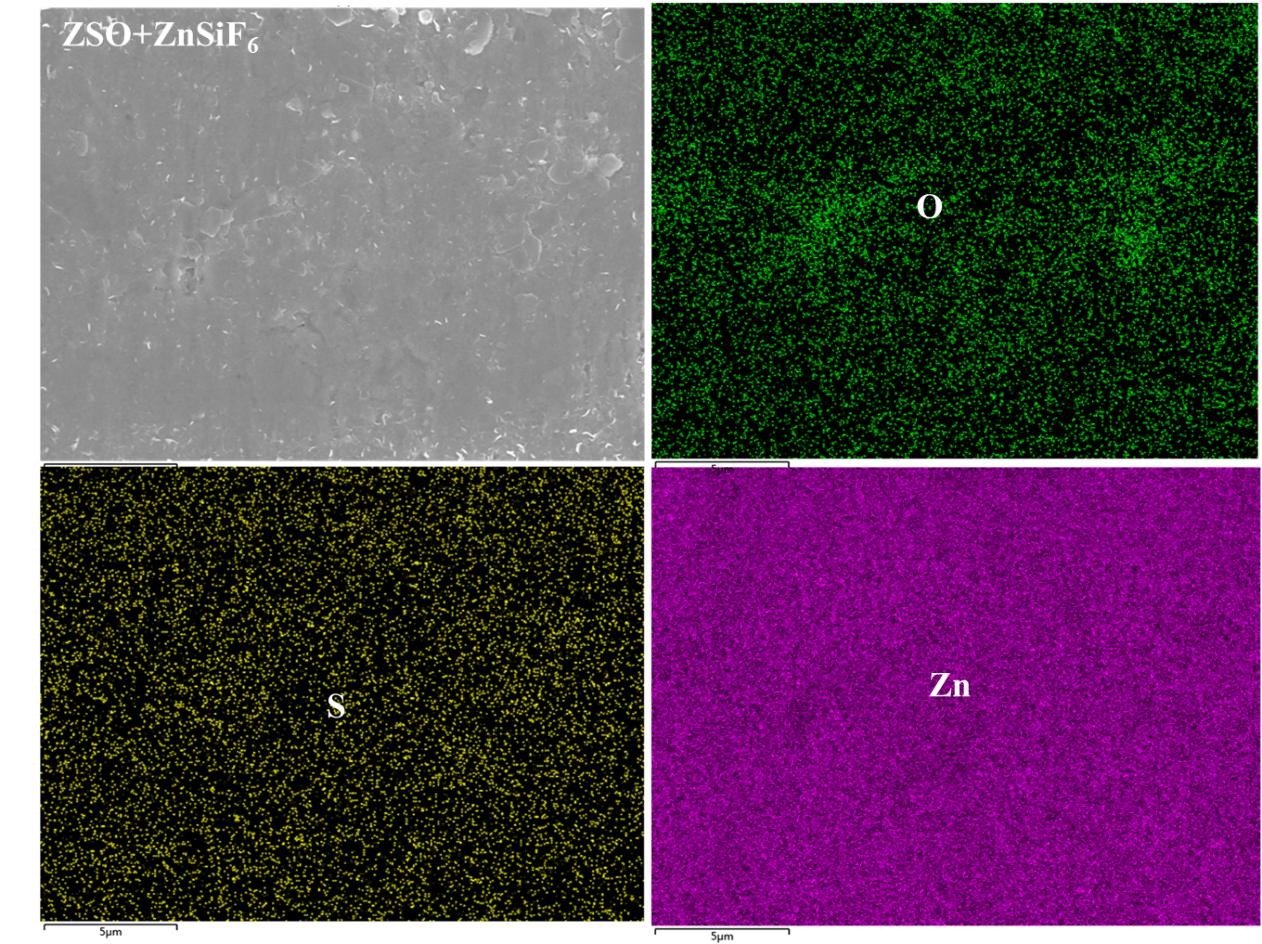


**Figure S20.** SEM-Mapping of Zn anode after 20 cycles in the ZSO-ZnSiF_6_ electrolyte.
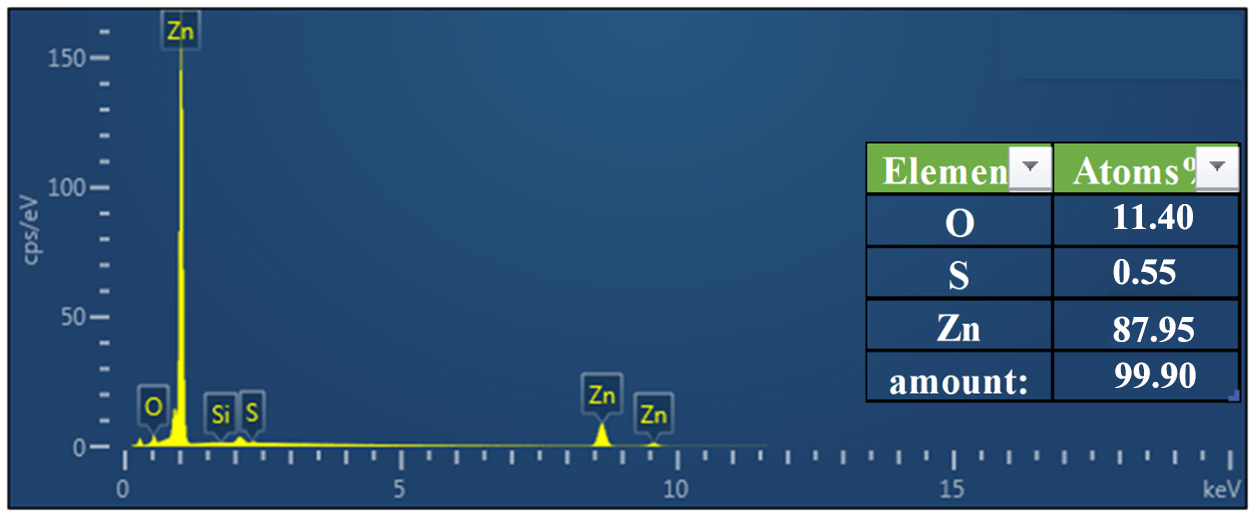
**Figure S21.** SEM-EDS of Zn anode after 20 cycles in the ZSO-ZnSiF_6_ electrolyte.


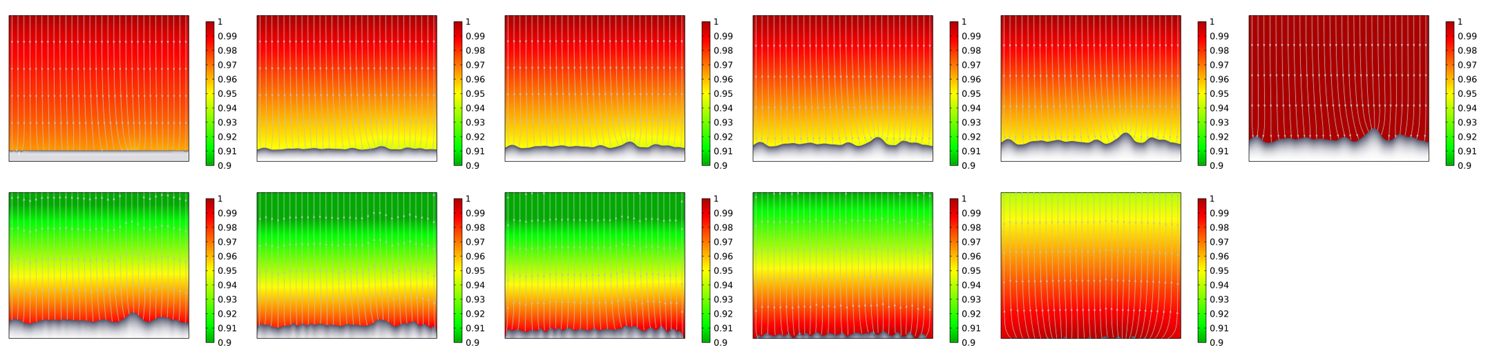


**Figure S22.** COMSOL simulation of Zn corrosion and HER during Zn plating/stripping process in the ZSO electrolyte.


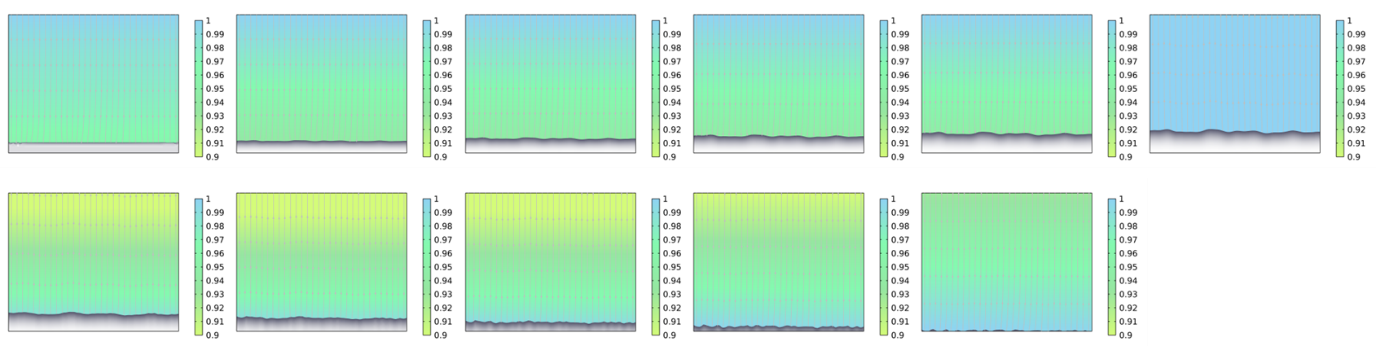


**Figure S23.** COMSOL simulation of Zn corrosion and HER during Zn plating/stripping process in the ZSO-ZnSiF_6_ electrolyte.


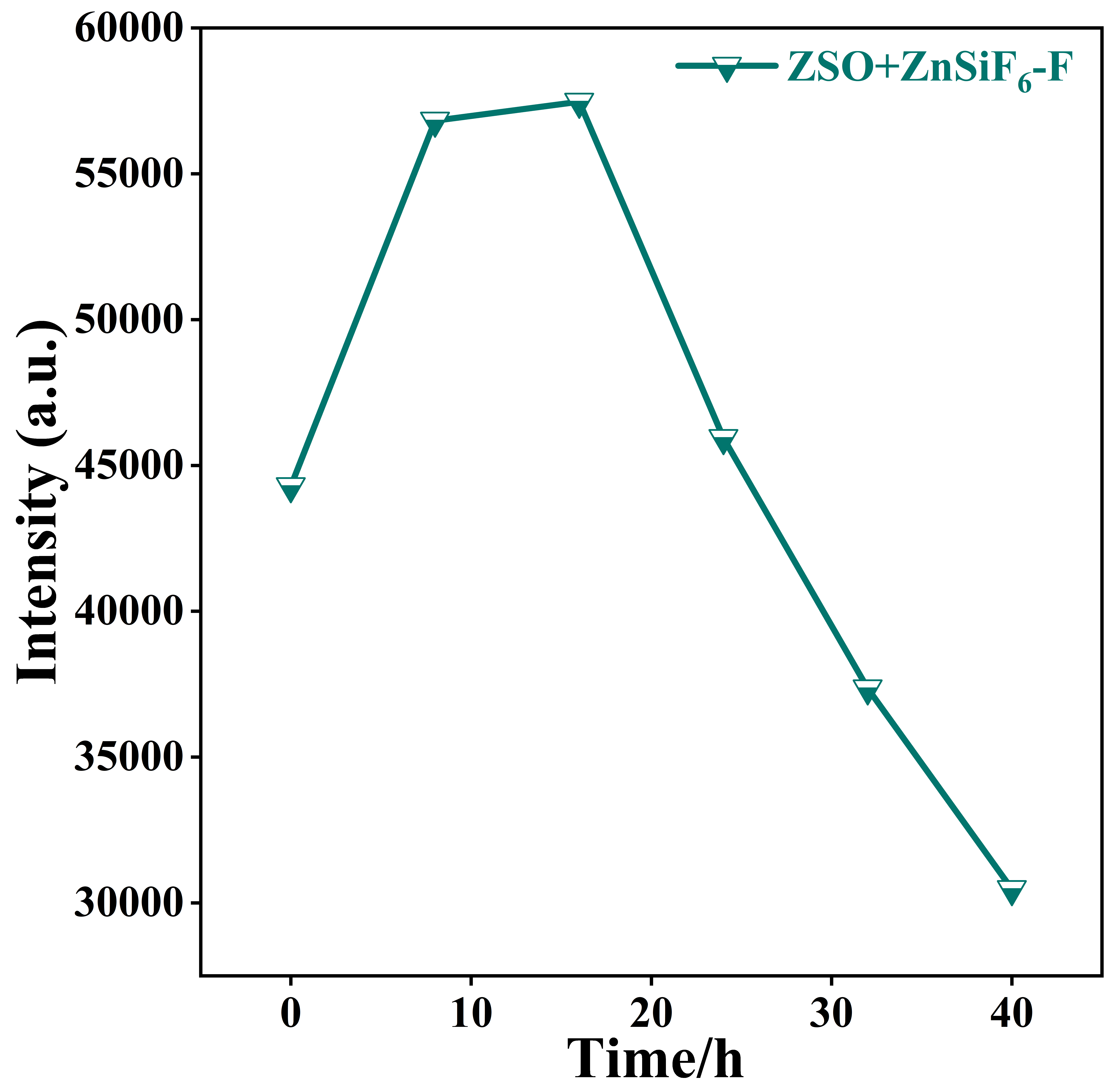


**Figure S24.** The depth distribution of F on the Zn anode after 20 cycles in the ZSO-ZnSiF_6_ electrolyte.
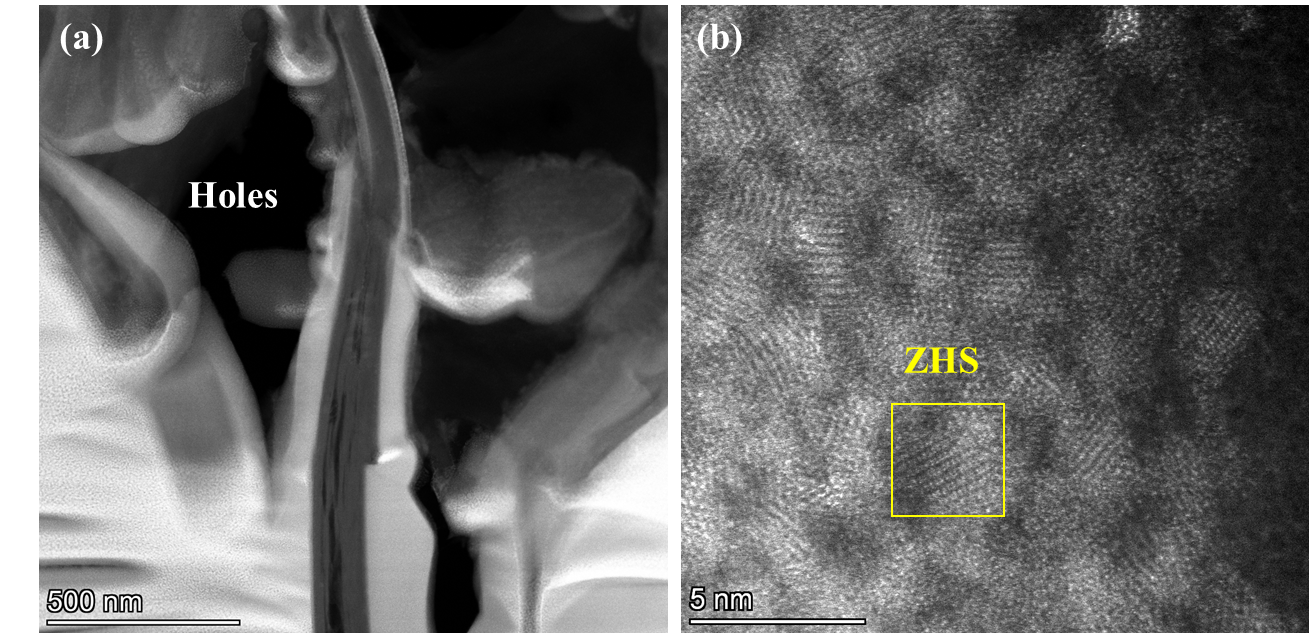
 **Figure S25.** STEM and HAADF images of the Zn anode after 20 cycles in the ZSO electrolyte.


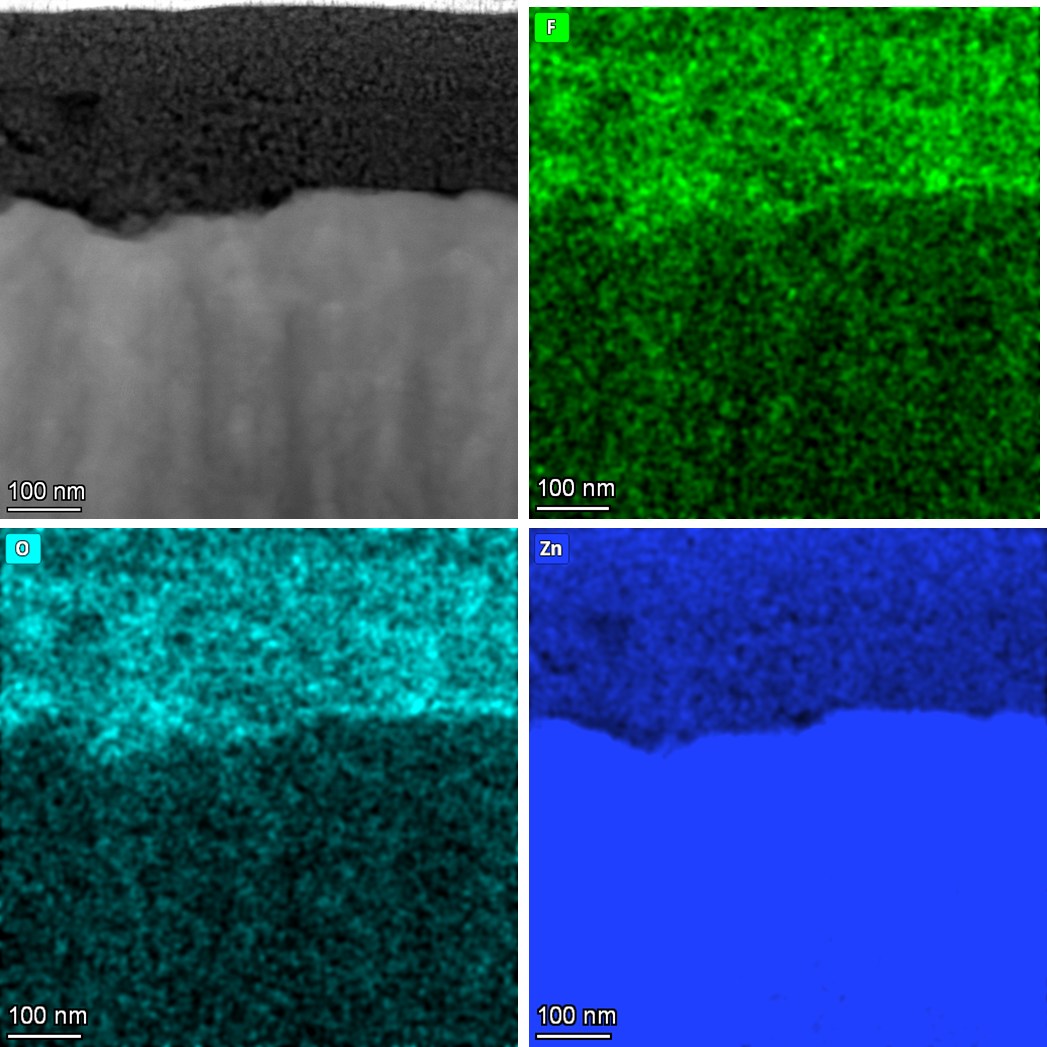


**Figure S26.** STEM and mapping image of SEI after 20 cycles in the ZSO-ZnSiF_6_ electrolyte.


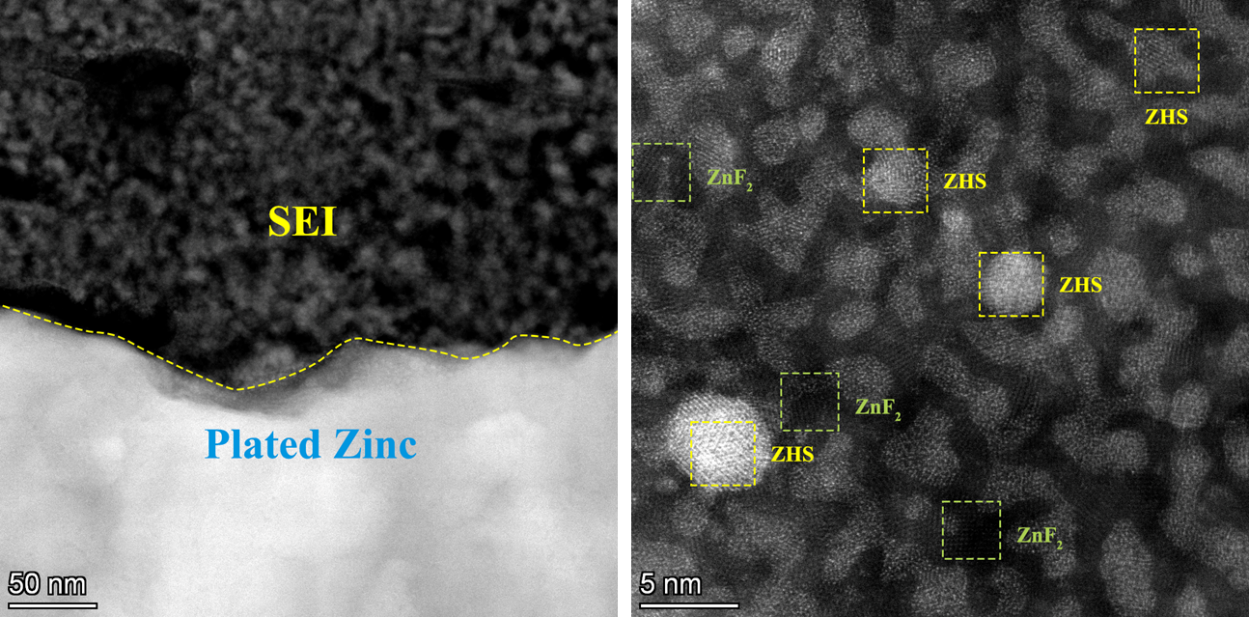


**Figure S27** STEM and HAADF images of the Zn anode after 20 cycles in the ZSO-ZnSiF_6_ electrolyte.


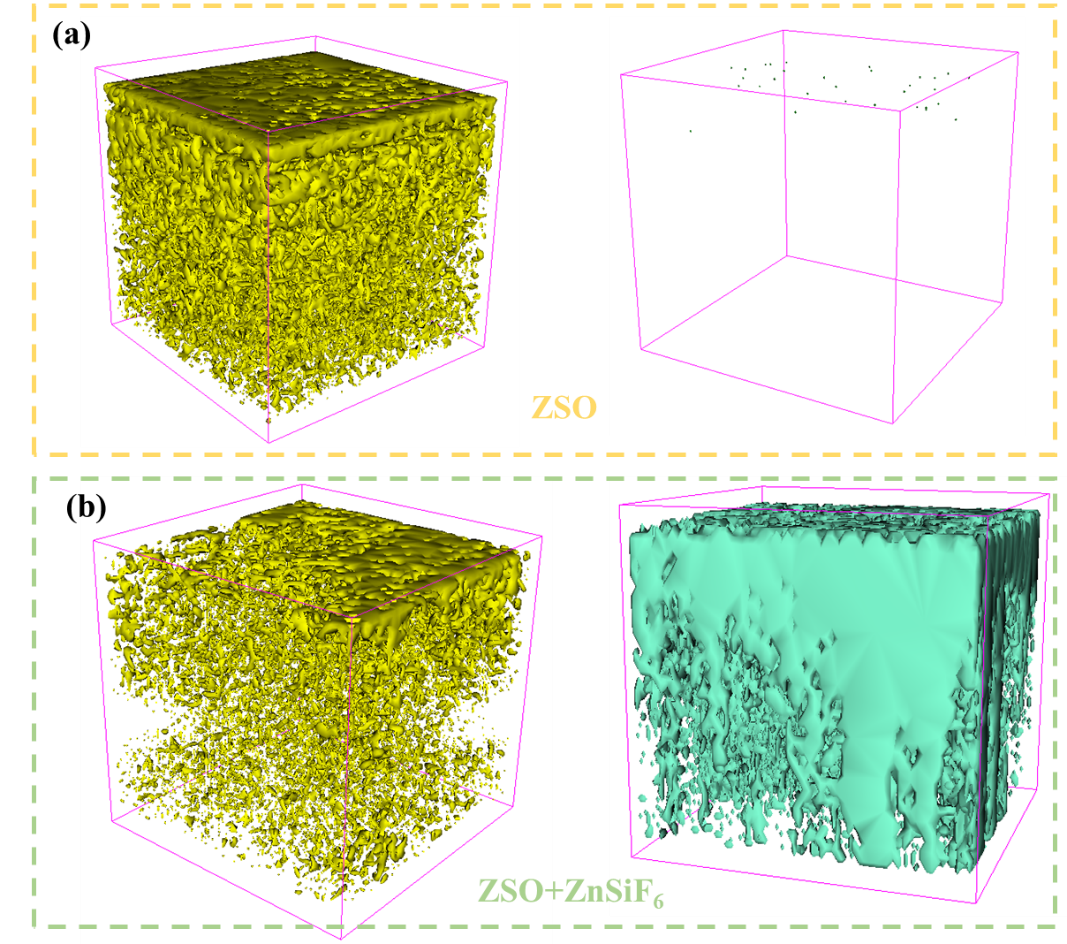


**Figure S28.** TOF-SIMS 3D render images of Zn anode after 20 cycles in the ZSO and ZSO-ZnSiF_6_ electrolyte.


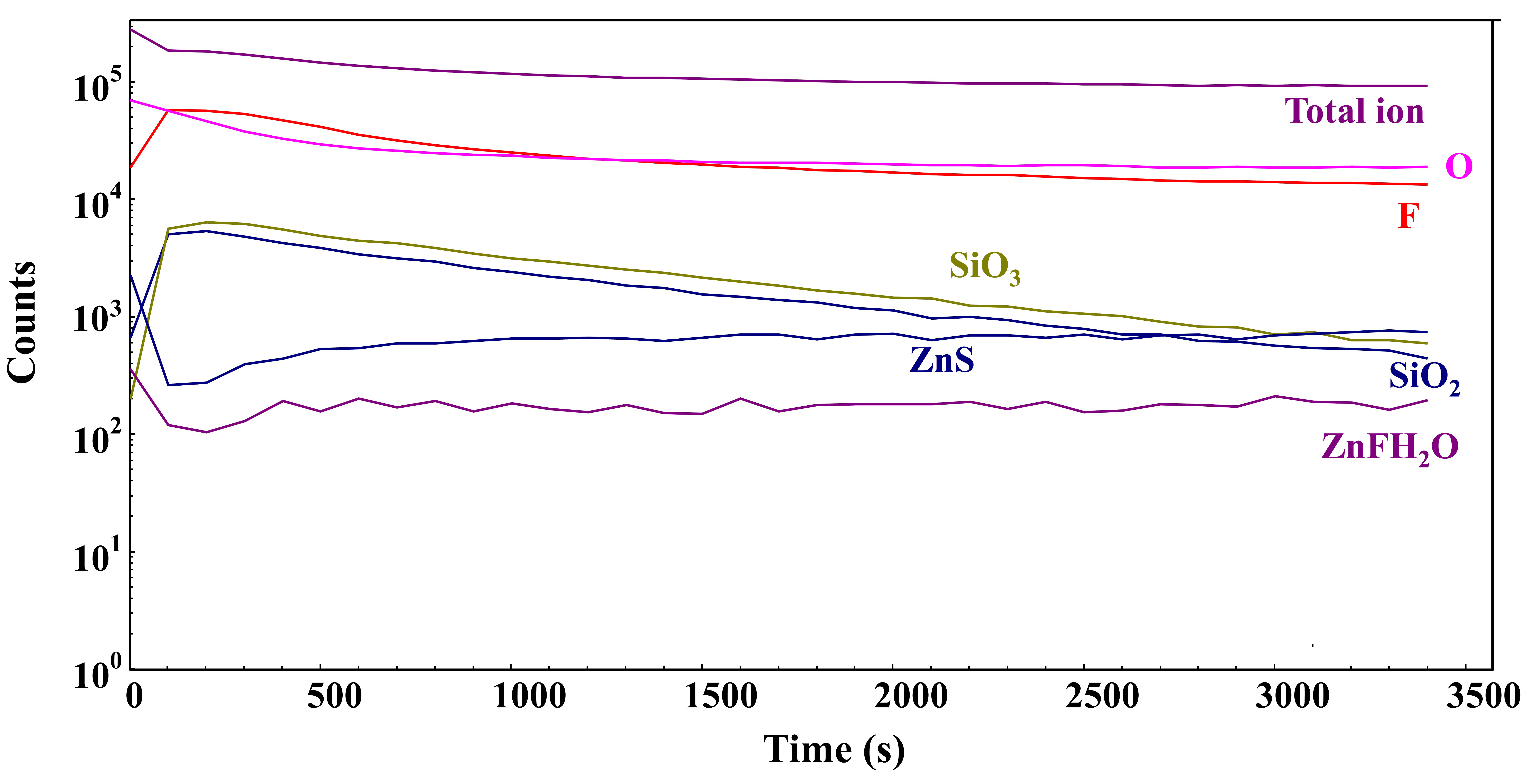


**Figure S29**. Depth profile of the elements on the surface of Zn anode after 20 cycles in the ZSO-ZnSiF_6_ electrolyte as determined by ToF-SIMS analysis.

**Table S1**. Simulated Data of Rct for the ZSO Electrolyte.

| **T (℃)** | **1000/T** | **Rct** | **Ln(1/Rct)** |  |  |  |
| --- | --- | --- | --- | --- | --- | --- |
| 30 | 3.300330033 | 328.3 | -5.793927825 |  |  |  |
| 40 | 3.194888179 | 203.7 | -5.316648323 |  |  |  |
| 50 | 3.095975232 | 131.4 | -4.878246106 |  |  |  |
| 60 | 3.003003003 | 86.38 | -4.458756168 |  |  |  |
| 70 | 2.915451895 | 47.11 | -3.852485293 |  |  |  |

**Table S2**. Simulated Data of Rct for the ZSO-ZnSiF_6_ Electrolyte.

| **T (℃)** | **1000/T** | **Rct** | **Ln(1/Rct)** |  |  |  |
| --- | --- | --- | --- | --- | --- | --- |
| 30 | 3.300330033 | 647.6 | -6.473273222 |  |  |  |
| 40 | 3.194888179 | 488.6 | -6.191544159 |  |  |  |
| 50 | 3.095975232 | 301.5 | -5.708770016 |  |  |  |
| 60 | 3.003003003 | 210.6 | -5.3499606 |  |  |  |
| 70 | 2.915451895 | 114.4 | -4.739701079 |  |  |  |

**References**

[1] Y. Huang, H. Yan, W. Liu, F. Kang, *Angewandte Chemie International Edition* **2024**, e202409642, <https://doi.org/10.1002/anie.202409642>.

[2] a) H. Sun, P. Ren, J. R. Fried, *Computational and Theoretical Polymer Science* **1998**, *8* (1-2), 229, <https://doi.org/10.1016/s1089-3156(98)00042-7>; b) H. Sun, *The Journal of Physical Chemistry B* **1998**, *102* (38), 7338, <https://doi.org/10.1021/jp980939v>.

[3] O. Borodin, M. Olguin, P. Ganesh, P. R. C. Kent, J. L. Allen, W. A. Henderson, *Physical Chemistry Chemical Physics* **2016**, *18* (1), 164, <https://doi.org/10.1039/c5cp05121e>.

[4] Y. Yang, H. Hua, Z. Lv, M. Zhang, C. Liu, Z. Wen, H. Xie, W. He, J. Zhao, C. C. Li, *Advanced Functional Materials* **2022**, *33* (10), 2212446, <https://doi.org/10.1002/adfm.202212446>.

[5] G. Kresse, D. Joubert, *Physical Review B* **1999**, *59* (3), 1758, <https://doi.org/10.1103/PhysRevB.59.1758>.

[6] a) J. Klimeš, D. R. Bowler, A. Michaelides, *Journal of Physics: Condensed Matter* **2010**, *22* (2), <https://doi.org/10.1088/0953-8984/22/2/022201>; b) *Physical Review B* **2011**, *83* (19), 195131, <https://doi.org/10.1103/PhysRevB.83.195131>.

[7] a) Y. Guo, W. Cai, Y. Lin, Y. Zhang, S. Luo, K. Huang, H. Wu, Y. Zhang, *Energy Storage Materials* **2022**, *50*, 580, <https://doi.org/10.1016/j.ensm.2022.06.001>; b) C. Li, X. Xie, H. Liu, P. Wang, C. Deng, B. Lu, J. Zhou, S. Liang, *National Science Review* **2022**, *9* (3), nwab177, <https://doi.org/10.1093/nsr/nwab177>; c) P. Sun, W. Liu, D. Yang, Y. Zhang, W. Xiong, S. Li, J. Chen, J. Tian, L. Zhang, *Electrochimica Acta* **2022**, *429*, 140985, <https://doi.org/10.1016/j.electacta.2022.140985>; d) H. Tian, J. L. Yang, Y. Deng, W. Tang, R. Liu, C. Xu, P. Han, H. J. Fan, *Advanced Energy Materials* **2022**, *13* (1), 2202603, <https://doi.org/10.1002/aenm.202202603>; e) X. Zeng, J. Mao, J. Hao, J. Liu, S. Liu, Z. Wang, Y. Wang, S. Zhang, T. Zheng, J. Liu, P. Rao, Z. Guo, *Adv Mater* **2021**, *33* (11), e2007416, <https://doi.org/10.1002/adma.202007416>; f) X. Zhao, X. Zhang, N. Dong, M. Yan, F. Zhang, K. Mochizuki, H. Pan, *Small* **2022**, *18* (21), e2200742, <https://doi.org/10.1002/smll.202200742>; g) Y. Zhong, Z. Cheng, H. Zhang, J. Li, D. Liu, Y. Liao, J. Meng, Y. Shen, Y. Huang, *Nano Energy* **2022**, *98*, 107220, <https://doi.org/10.1016/j.nanoen.2022.107220>; h) J. Zhou, M. Peng, X. Xia, S. Qian, Z. Wang, C. Zhu, X. Zeng, H. Ji, S. Wang, X. Zhou, J. Liu, X. Shen, Y. Cheng, T. Qian, C. Yan, *Nano Letters* **2022**, *22* (7), 2898, <https://doi.org/10.1021/acs.nanolett.2c00065>; i) J. Zhou, F. Wu, Y. Mei, Y. Hao, L. Li, M. Xie, R. Chen, *Adv Mater* **2022**, *34* (21), e2200782, <https://doi.org/10.1002/adma.202200782>.
